# *In-vitro* Recordings of Neural Magnetic Activity From the Auditory Brainstem Using Color Centers in Diamond: A Simulation Study

**DOI:** 10.3389/fnins.2021.643614

**Published:** 2021-05-13

**Authors:** Mürsel Karadas, Christoffer Olsson, Nikolaj Winther Hansen, Jean-François Perrier, James Luke Webb, Alexander Huck, Ulrik Lund Andersen, Axel Thielscher

**Affiliations:** ^1^Department of Health Technology, Technical University of Denmark, Kongens Lyngby, Denmark; ^2^Department of Neuroscience, Faculty of Health and Medical Sciences, University of Copenhagen, Copenhagen, Denmark; ^3^Department of Physics, Technical University of Denmark, Kongens Lyngby, Denmark; ^4^Danish Research Centre for Magnetic Resonance, Centre for Functional and Diagnostic Imaging and Research, Copenhagen University Hospital Hvidovre, Hvidovre, Denmark

**Keywords:** cable equation, neural magnetic field, magnetometry, NV centers, Monte Carlo, optogenetics, fiber optics, Kubelka-Munk model

## Abstract

Magnetometry based on nitrogen-vacancy (NV) centers in diamond is a novel technique capable of measuring magnetic fields with high sensitivity and high spatial resolution. With the further advancements of these sensors, they may open up novel approaches for the 2D imaging of neural signals *in vitro*. In the present study, we investigate the feasibility of NV-based imaging by numerically simulating the magnetic signal from the auditory pathway of a rodent brainstem slice (ventral cochlear nucleus, VCN, to the medial trapezoid body, MNTB) as stimulated by both electric and optic stimulation. The resulting signal from these two stimulation methods are evaluated and compared. A realistic pathway model was created based on published data of the neural morphologies and channel dynamics of the globular bushy cells in the VCN and their axonal projections to the principal cells in the MNTB. The pathway dynamics in response to optic and electric stimulation and the emitted magnetic fields were estimated using the cable equation. For simulating the optic stimulation, the light distribution in brain tissue was numerically estimated and used to model the optogenetic neural excitation based on a four state channelrhodopsin-2 (ChR2) model. The corresponding heating was also estimated, using the bio-heat equation and was found to be low (<2°C) even at excessively strong optic signals. A peak magnetic field strength of ∼0.5 and ∼0.1 nT was calculated from the auditory brainstem pathway after electrical and optical stimulation, respectively. By increasing the stimulating light intensity four-fold (far exceeding commonly used intensities) the peak magnetic signal strength only increased to 0.2 nT. Thus, while optogenetic stimulation would be favorable to avoid artefacts in the recordings, electric stimulation achieves higher peak fields. The present simulation study predicts that high-resolution magnetic imaging of the action potentials traveling along the auditory brainstem pathway will only be possible for next generation NV sensors. However, the existing sensors already have sufficient sensitivity to support the magnetic sensing of cumulated neural signals sampled from larger parts of the pathway, which might be a promising intermediate step toward further maturing this novel technology.

## Introduction

Negatively charged nitrogen-vacancy (NV) color centers in diamond are a novel tool to sense weak magnetic fields with nano- to millimeter spatial resolution, reaching sensitivity levels of pT/Hz^1/2^ and below even at low frequency ranges and under ambient temperatures (Taylor et al., 2008; Fescenko et al., 2020; Zhang et al., 2021). For this reason the technique has been suggested to be well suited for *in vitro* imaging of the neural activity of cultured nerve cells or of groups of nerves in brain slices (Hall et al., 2012). The sensing protocols are based on optically detected magnetic resonance (ODMR) in which external magnetic fields cause changes in the optically induced NV fluorescence. This method has recently been used to detect the magnetic field of living cells (Le Sage et al., 2013) and large axons (Barry et al., 2016), as well as to characterize the current flow in graphene structure (Tetienne et al., 2017).

**FIGURE 1:**
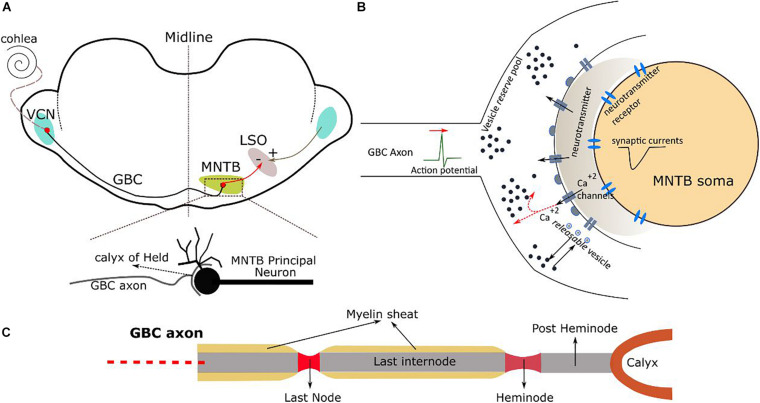
**(A)** Auditory brainstem pathway from VCN to MNTB and the calyx of Held synapse. MNTB cells project further to the lateral superior olive (LSO). **(B)** A single calyx of Held synapse connected to the soma of a MNTB principal cell. **(C)** The terminal part of a GBC axon and its different sections.

Traditional *in vitro* electrophysiological techniques allow recordings of the transmembrane voltage or current which helps to interpret the underlying mechanism of neural activity. However, their insufficient spatial sampling has led to the development of multi-electrode arrays (MEA) (Obien et al., 2015) or voltage-sensitive dye imaging (VSDI) (Stepan et al., 2015). VSDI, however, require complex addition of dyes which may not be suitable for all systems and has poor signal to noise ratio (Homma et al., 2009), and the spatial resolution of MEA is limited by the electrode density in the array (Obien et al., 2015). As higher spatiotemporal resolution and coverage is desired, NV-based magnetometry might offer an interesting, stain-free, alternative for the spatially resolved wide-field imaging of neural magnetic fields. Using NV-based magnetometry any sample which produces electric currents can in principle be imaged with high spatiotemporal resolution, without the need for genetic manipulation or staining. This method is completely passive and a sample can simply be placed on to a diamond sensor without any alterations to it, and can furthermore offer multi-modal capabilities, such as being used to sense electric fields (Dolde et al., 2011) and temperature (Acosta et al., 2010). For more detailed comparisons of NV-based magnetometry and existing alternatives for imaging neural signals please refer to e.g., (Schirhagl et al., 2014) and (Hall et al., 2012). We previously investigated the NV-based magnetometry method for imaging magnetic fields emerging from pyramidal cells in the gray matter of rodent hippocampal slices (Karadas et al., 2018). Complementing these findings, our aim here is to explore the feasibility of using NV-based magnetometry to also measure fast action potentials traveling along white matter pathways. Using detailed simulations, we analyze the electromagnetic fields generated by synchronous neural activity of the auditory pathway from the ventral cochlear nucleus (VCN) to the medial nucleus of the trapezoid body (MNTB) in mouse brainstem slices (Figure 1). This pathway provides a fruitful test case as (i) it is constituted by a dense bundle of hundreds of large-diameter axons which arise from the globular bushy cells (GBCs) in the VCN (Ford et al., 2015), (ii) the pathway can accommodate high frequency (∼200–400 Hz) firing (Taschenberger and von Gersdorff, 2000; Lorteije et al., 2009), thus allowing more averaging trials to increase signal to noise ratio (SNR), and (iii) the pathway is well suited to image action potentials in isolation without strong contributions from other neural signals. It is also a highly interesting target for neuroscience research due to the large size of GBC synaptic terminals, the Calyx of Held, which makes contact with principal cells from the MNTB. This is the only mammalian synapse allowing for simultaneous patch-clamp recording of the post- and pre-synaptic activities (Barnes-Davies and Forsythe, 1995; Borst et al., 1995). Most of the knowledge of pre-synaptic calcium dynamics and short-term synaptic plasticity comes from this synapse. Neurons from the MNTB project to the lateral superior olive (LSO), which integrates auditory inputs from both ears and plays a pivotal role for interaural discrimination (Joris and Trussell, 2018). Interestingly, both pre- and post-synaptic neurons from the Calyx of Held express the calcium binding protein parvalbumin (PV) (Felmy and Schneggenburger, 2004). Thus, by using PV as a promoter for Cre-dependent expression of light sensitive cation channel channelrhodopsin-2 in these neuronal populations, it should be possible to stimulate VCN and MNTB neurons with light.

In the present study, we simulate the extracellular magnetic fields created by neural activity traveling along this pathway in an *in vitro* preparation of a mouse brainstem slice. Our aim is to characterize the spatial and temporal features of the neural magnetic signals that would be sensed by an underlying planar NV sensor in order to assess the feasibility of this new imaging approach. Specifically, we model the dynamic responses of the GBCs and the principal cells of the MNTB to external stimulation and the synaptic transmission between both in NEURON (Hines and Carnevale, 2001) using previously reported morphological and biophysical characteristics (Lorteije et al., 2009). In addition to simulating the responses to the well-established electric stimulation, we explore the use of optogenetic stimulation as a means to prevent interferences of the stimulation with the magnetic measurements (Gunaydin et al., 2010; Deisseroth, 2011). Our simulations allow estimations of the expected magnetic field strengths as well as the required sensitivity level of the imaging system.

## Materials and Methods

### Cell Models of the GBC-MNTB Pathway

The axons of the globular bushy cells project from the VCN to the principal neurons in the contralateral MNTB (Figure 1A), where they end in large axo-somatic excitatory glutamatergic synapses that are termed calyces of Held (Figure 1B). The principal neurons from the MNTB project mainly to the lateral superior olive (LSO). Most GBC axons form only one calyx that connects to a single principal cell. Thus, there are one-to-one connections between globular bushy cells and their corresponding MNTB principal cells (Borst and Soria van Hoeve, 2012). In the following, we describe the kinetic and geometric models employed in our simulations.

#### Globular Bushy Cells (GBC)

Each globular bushy cell is modeled by five different components: Soma, dendrites, axon hillock, initial segment, and myelinated axon (Xie and Manis, 2013; Manis and Campagnola, 2018). The soma is a sphere with a diameter of 20 μm. A primary dendrite of 50 μm length and 3 μm diameter connects to the soma, and two secondary dendrites branch from the distal part of the primary dendrite to the right and left with lengths of 50 μm and diameters of 2 μm. The morphological data of the axon is taken from Ford et al. (2015). The axon hillock is oriented 90° with respect to the primary dendrite and has 15 μm length and a maximal diameter of 2.5 μm and then decreases to a diameter of 1 μm for the initial segment. The myelinated axon has a tapered structure, so that its diameter increases from 3 μm at the initial segment to ∼3.3 μm when close to the synaptic terminals. Similarly, the node diameters increase from 1.5 to 2 μm close to the synaptic terminals. The internode length initially remains constant at ∼200 μm, but then systematically decreases on the last ∼800 μm of the axon and reaches ∼40 μm close to the synaptic terminals. The length of all nodes is 1 μm and constant. These systematic variations in the axon morphology increase the maximum depolarization of the nodes close to the calyx of Held and aids transmission of action potentials. The calyx itself is modeled as a half ring (diameter of 13 μm, thickness of 2 μm), unless otherwise stated. Please see the next section for a detailed description of the axon pathway.

The cell membrane of the soma, primary dendrites and initial segment contain inactivating Na^+^ channels, low-threshold K^+^ (K_*LT*_) and high-threshold K^+^ (K_*HT*_) channels, and hyperpolarization-activated cation (I_*h*_) channels with the kinetics taken from Rothman and Manis (2003). The corresponding relations between the ionic currents and their kinetics, including the channel properties, are given in Supplementary Paragraph 1.

In order to model the cell membrane of the internodes of the axon, the passive myelinated axon model C from Richardson et al. (2000) was implemented in NEURON. The periaxonal space is not included in the model and it was further assumed that the myelin sheath membrane has constant conductivity (Supplementary Paragraph 2 contains detailed information). The nodes of Ranvier of the axon are modelled as containing inactivating Na^+^ and low-threshold K^+^ (KLT) channels with Hodgkin–Huxley-like kinetics as well as a leak conductance (Rothman and Manis, 2003). The last internode is followed by a longer node called heminode, which has the same channel types and densities as the other nodes of Ranvier (Ford et al., 2015). The heminode connects to the calyx of Held via a short internode named post-heminode, which we included in the modal as a capacitor without any conductance.

The membrane of the calyx of Held is modelled as having high-threshold K^+^ (KHT) and hyperpolarization-activated cation (h) channels and a voltage sensitive calcium conductance (Figure 1B; Supplementary Paragraph 1 contains details) (Borst and Sakmann, 1998).

##### Synaptic connections

The synaptic transmission model consists of two kinetics: Vesicle release and AMPA receptor. The vesicle release mechanism is modeled by 500 independent active release zones each with one readily releasable vesicle (Graham et al., 2001). The mechanism is activated by arrival of an action potential (AP) to the calyx of Held. It initiates fast and slow calcium transients for 1 and 2 ms, respectively. The fast calcium transient activates opening of the fast and slow gates. The fast gates go to open state quickly upon arrival of the AP and close rapidly. On the other hand, slow gates open partially and close slowly so that a potentiation can occur by a following AP. A release probability for all active zones is determined by the product of open states of slow and fast gates. Once a vesicle is released, a 1 ms square pulse of neurotransmitter T is produced that drives a corresponding AMPA receptor-mediated EPSC. The receptors are placed at the soma and partially at the axon initial segment of the principal cell. A 6-state gating model is used to simulate the AMPA receptor kinetics, composed of three closed states, fast and slow open states, and one desensitized state (Raman and Trussell, 1992). At the same time, the vesicles are replenished by a constant background replenishment from a large reserve pool and enhanced replenishment driven by the slow (residual) calcium transient. Further details on the kinetic model and parameters can be found in Supplementary Section 4.

#### MNTB Principal Cells

A principal neuron has a simple spherical perikaryon, one or two short and thin dendritic trees (Smith et al., 1998), and its axon provides inhibitory input to the LSO. Here, it is modeled by three compartments: Soma, dendrite, and axon. The soma is a sphere with a diameter of 20 μm. The dendrite consists of cylinders of 50 μm length and 3 μm diameter and makes 2 branches of 10 μm at the end. The axon is oriented 90° with respect to the dendrite and composed of 5 internodes and nodes. Each internode and node have constant diameters of 2 and 1 μm, respectively, and lengths of 100 and 1 μm. As for the axonal pathways of the GB cells (see next section), the axon pathway is generated by a spline interpolation of randomly generated five points on the way to the LSO. The model has Hodgkin–Huxley-like dynamics with a high density of Na^+^, low-threshold K^+^ and high-threshold K^+^ channels, similar to a previous model of the MNTB neuron (Wang et al., 1998). Whereas low-threshold K^+^ channels dampen excitability so that the calyceal inputs generates a single AP, high-threshold K^+^ channels guarantee rapid gating and shorten the AP (Brew and Forsythe, 1995). Details on the ion channel dynamics of the principal cells can be found in Supplementary Section 3.

### Morphological Model of the GBC-MNTB Axonal Pathway

**FIGURE 2:**
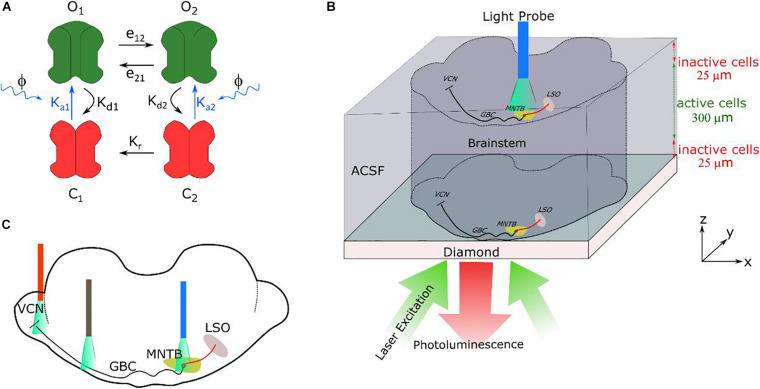
**(A)** Four-state model, with two open and two closed states, of channelrhodopsin-2 (ChR2) as described in Nikolic et al. (2009). e12, e21, Kd1, Kd2, Kr are the rate constants (Supplementary Table 5) and Ka1 and Ka2 are the light dependent constants. **(B)** Schematic illustration of the simulated brainstem slice placed on the diamond sample. It is assumed that the neural cells in a distance of up to 25 μm to the diamond are dysfunctional due to the preparation. The changes in photoluminescence emitted by the NV-layers in the diamond, caused by the neural magnetic fields, can be recorded using an arrangement like an inverted microscope, with the camera replacing the photodetector (Wojciechowski et al., 2018). In optical stimulation, a light probe is placed on the MNTB (drawing is not to scale). **(C)** Three different illumination positions along at the pathway were simulated (VCN region containing the soma of the GB cells, the myelinated axons of the GB cells and the MNTB region) to test the feasibility of light excitation.

The white matter pathway from VCN to MNTB is reconstructed from the confocal images of transverse brainstem slices given in Ford et al. (2015) and Kronander et al. (2017). Here, the thickness of the brainstem slice is modelled as 350 μm, consisting of a bottom layer of 25 μm of inactive cells (affected by the cutting procedure), a 300 μm thick layer of active cells and a top layer of 25 μm of inactive cells (Figure 2B). For each globular bushy cell, the axonal path is determined by spline interpolation of eight points as follows:

(i). The MNTB area is defined as an elliptical region (semi-major axis 200 μm and semi-minor axis 100 μm, active cell thickness 300 μm) which is 400 μm away from the brain midline (Weatherstone et al., 2017). A random position (x_*c*_, y_*c*_, z_*c*_) is chosen in this area as the center location of the calyx of Held. This position is also the center of the soma of the corresponding principal cell.
(ii). Seven rectangular areas (each with 25 × 25 μm^2^) along the axonal pathway were manually predefined. A random position is picked from within each area, and the eighth point is added at (x_*c*_, 0, 0) for the calyx of Held.
(iii). The axonal pathway is generated by spline interpolation of the eight points, and the complete pathway including the GBC soma is finally moved by y_*c*_ in y-direction and by z_*c*_ along z-direction.
(iv). The soma of the principal cell is placed at (x_*c*_, y_*c*_, z_*c*_) and rotated around its z-axis by a random angle between -π/2 and π/2. The calyx of Held is also rotated randomly around its z-axis.

In order to model a brainstem stem slice with a thickness of 300 μm, in total 300 cells are created. In the literature, the total number of principal cells is given rather than the neural density, and the average number of cells per MNTB area is estimated to be around 2,500 in mice (Weatherstone et al., 2017). Considering 8–10 slices per brainstem and a total height of the VCN and MNTB area of around 3,000 μm, we assume to have around 300 cells in a 300 μm thick slice through the central part of the MNTB area as a worst case estimate. However, it is more likely that all the calyces present within 600 μm which results in ∼1,250 cells per slices (Kronander et al., 2017). We therefore report both the results for the worst case and the more likely case in the following.

The neuronal density also varies for different slices and species. Therefore, our results should be linearly rescaled to match those for a different neuron density.

### Calculation of the Neural Magnetic Field and Electric Potential

It is known that the axial and transmembrane currents are the major sources of the magnetic field and electric potentials, respectively (Woosley et al., 1985). Therefore, we follow the two step approach given in Karadas et al. (2018) to determine the extracellular neural magnetic fields and electric potential: Initially, the membrane potentials V_*m*_^*n*^ and transmembrane currents I_*m*_^*n*^ of the simulated neuron are calculated using the NEURON software package (v7.4) (Hines and Carnevale, 2001) with a time resolution of 25 μs. Then, the extracellular magnetic fields B and the electric potential φ at the sensor surface are determined from the simulations using a forward modeling scheme for the simplified case of an unbounded homogenous volume conductor, as described in Karadas et al. (2018). Finally, the impact which the extracellular current flow in the non-simplified volume conductor exerts on the measured magnetic field is accounted for using a scaling factor as given in Karadas et al. (2018). This scaling factor approximates the effects of the conductivity anisotropy in the brain slice, the boundary between the brain slice and the underlying sensor, and the fluid surrounding the brain slice on the extracellular current flow.

### Modeling of Optogenetic Stimulation

Optogenetic stimulation might be a good alternative to the well-established electric stimulation to avoid interferences of the stimulation with the magnetic measurements. To investigate the efficiency of optogenetic stimulation to generate APs across all axons of the pathway, we implemented the “light-neuron” model given in Foutz et al. (2012). The model simulates the neural dynamic in brain tissue generated by an optical probe which couples to light-sensitive neurons. The latter were modeled using the cable equation, extended by Channelrhodopsin-2 (ChR2) channels. In the following, we describe the ChR2 channel model and the light distribution in tissue created by an optical probe. In addition, the model used to estimate the thermal effects due to light absorption is outlined.

#### ChR2 Photo-Current Modeling

Channelrhodopsin-2 (ChR2) was modeled with two closed (C_1_, C_2_) and two open (O_1_, O_2_) channel states (Figure 2A; Nikolic et al., 2009; Foutz et al., 2012). The initial state of a channel is C_1_. Under illumination, C_1_transits into open state O_1_ at a rate constant of *K*_*a1*_. A channel in O_1_ state exponentially decays to closed state C_2_ at a rate K_*d1*_, or instead transits to the second open state O_2_ at a rate constant of e_12_. State O_2_ is more stable and has lower ion conductance with respect to O_1_. The O_2_ state can exponentially decay to C_2_ at a rate K_*d1*_ or return to O_1_ at a rate constant of *e*_21_. Channels being in C_2_ state can go into O_2_ state at an exponential increase rate *K*_*a*2_,or they go to C_1_ state by a slow thermal conversion with exponential decay rate K_*r*_. The instantaneous rate of change of these states was defined by a set of rate equations (Grossman et al., 2011b):

(1) $$\frac{d\,O_{1}}{d\,t}=K_{a\,1}\,C_{1}-\left(K_{d\,1}+e_{12}\right)\,O_{1}+e_{21}\,O_{2}$$

(2) $$\frac{d\,O_{2}}{d\,t}=K_{a\,2}\,C_{2}+e_{12}\,O_{1}-\left(K_{d\,2}+e_{21}\right)\,O_{2}$$

(3) $$\frac{d\,C_{2}}{d\,t}=K_{d\,2}\,O_{2}-\left(K_{a\,2}+K_{r}\right)\,C_{2}$$

(4) $$C_{1}+C_{2}+O_{1}+O_{2}=1$$

In this state model, only the rate constants K_*a1*_ and K_*a2*_ that control the transition from the closed to the open states are dynamically changed by the light stimulation:

(5) $${\text{K}}_{\text{ai}}=\left\{ \begin{array}{cc} \varepsilon_{\text{i}}\,\phi \,\left(1-{\text{e}}^{-\frac{\text{t}-{\text{t}}_{\text{ON}}}{\tau }}\right)\; & \mathrm{during}\,\mathrm{illumination}\\ \varepsilon_{\text{i}}\,\phi \,\left({\text{e}}^{-\frac{\text{t}-{\text{t}}_{\text{OFF}}}{\tau }}-{\text{e}}^{-\frac{\text{t}-{\text{t}}_{\text{ON}}}{\tau }}\right)\; & \mathrm{after}\,\mathrm{illumination} \end{array}\right.$$

Here, ε_*i*_ is the quantum efficiency of channelrhodopsin, ϕ is the photon flux per area during illumination. Once the irradiance is determined for each ChR2 molecule, ϕ is calculated by considering the cross-sectional area of the molecule (∼1.2 × 10^–8^ μm^2^) (Hegemann et al., 2005) and the wavelength of the light (473 nm). τ is the time constant of the channel. *t*_*ON*_ and *t*_*OFF*_ are the time points at which illumination is switched on and off. Once O_1_ or O_2_ are reached, the photocurrent through the membrane for illumination ON is calculated as

(6) $$I_{C\,h\,R\,2}=i_{m\,a\,x}\,\left(O_{1}+\gamma \,O_{2}\right)\,\frac{\left(1-e^{-U/U_{0}}\right)}{U/U_{1}},$$

where *i*_*max*_ = (*V*−*E*_*ChR*2_)*g*_*ChR*2_ is the maximum reachable ChR2 current depending on the reversal potential E_*ChR2*_ (set to 0 mV) and the channel conductance per area (g_*ChR2*_ = g_1_ρ_*ChR2*_). Constant γ (set to 0.05) is the conductance ratio between O_1_ and O_2_. The last term in (6) accounts for an inwardly rectifying current–voltage curve as shown experimentally in Grossman et al. (2011b), where U is the absolute transmembrane potential. U_0_ and U_1_ are empirical constants set to 40 and 15 mV, respectively.

After the illumination goes off, the ChR2 current shows an exponential decay which is explained by a fast (i_*fast*_) and slow (i_*slow*_) component as given by

(7) $$i=i_{s\,l\,o\,w}\,e^{-\Lambda_{1}\,\left(t-t_{O\,F\,F}\right)}+i_{f\,a\,s\,t}\,e^{-\Lambda_{2}\,\left(t-t_{O\,F\,F}\right)},$$

where Λ_1_ and Λ_2_ are decay factors. The complete set of equations defining the ChR2 dynamics and parameters are provided in Supplementary Section 5.

#### Light Model

There are four main factors defining the light distribution in tissue: (1) The light distribution at the source, (2) the geometric spread, (3) the scattering, and (4) the absorbance by the medium. The most established approaches to determine the light distribution in tissue are the Kubelka-Munk (KM) model and Monte Carlo (MC) simulations. The KM approach provides an analytical expression so that the irradiance can be determined at any point and easily incorporated as input into the NEURON solver. In the KM model, the light intensity is described by the transmitted and the backscattered components which are defined by two coupled differential equations (Mobley et al., 2014). The light beam in the forward direction decreases in intensity due to the absorption and scattering processes and gains intensity from the backscattering process from photons being scattered in the reverse direction. In a cylindrical coordinate system, the irradiance I at a point is defined by I(r,z) = T(r,z) I_0_, where I_0_ is the irradiance of the source and T(r,z) is the transmittance at the radial distance r and depth z. The transmittance factor is wavelength dependent and can be represented by three linear components (Foutz et al., 2012)

(8) T⁢(r,z)=G⁢(r,z)⁢C⁢(z)⁢M⁢(r,z),

where G describes the distribution of light emitted by the light source, C describes the geometrical spreading of the light, and M describes the scattering and the absorbance of the light according to the KM model. G(r,z) is generally defined by a Gaussian function which takes into the account expansion from the source. The geometric spread function C(z) is calculated by the law of conservation of radiant power. Therefore, as the light travels it will diverge and its intensity decreases. For the radius R(z) of the light cone at depth of z starting from R_0_ emission, the spread function is:

(9) $$C\,\left(z\right)=\frac{R_{0}}{R\,\left(z\right)}$$

The solution for factor M is given by

(10) $$M\,\left(x\right)=\frac{b}{a\,s\,i\,n\,h\,\left(b\,\mu_{s}\,x\right)+b\,c\,o\,s\,h\,\left(b\,\mu_{s}\,x\right)}$$

with

(11) $$a=1+\frac{\mu_{a}}{\mu_{s}}\;\text{and}\;b\;\sqrt{a^{2}-1},$$

where the parameter $x=\sqrt{z^{2}+r^{2}}$ is the travelled distance, μ_*s*_ is the scatter coefficient per unit thickness (in [mm^–1^]), and μ_*a*_ is the absorption coefficient per unit thickness (in [mm^–1^]). The KM model relies on the simplifying assumptions of (1) isotropic scattering, which is valid in the diffusive regime for depths of multiple millimeters, and (2) isotropic illumination, thus neglecting the finite geometry and size of the illumination. In contrast, in optogenetics, scattering is highly anisotropic at the relevant distance scales and wavelengths and the light source typically has a comparable size to the tissue of interest (Yona et al., 2016). Here, we implemented the KM model using custom-written Python scripts.

For this reason, we additionally simulated the light distribution using the MC method, where the random walk of photon packets is traced through the tissue in three dimensions (Wang et al., 1995). In this method, photon packets are launched from a light source so that each packet has a random initial position and direction according to the geometry and numerical aperture of the source. Then, the light transport through the tissue is simulated as iterative process. Initially, a weight of 1 is given to each photon packet and it is moved by a random distance depending on the absorption and scattering coefficients. After each iteration, the photon packet loses weight by a factor of μ_*a*_/(μ_*a*_ + μ_*s*_), and this fraction is added to the total amount of light absorbed at the corresponding tissue position. The new trajectory is defined by an anisotropic scattering model using the Henyey–Greenstein (HG) phase function. This procedure is iterated for each photon packet until its weight decreases below a certain threshold. The details of the implementation are given in Jacques (2010). Here, we used the MC implementation that was made available by these authors.

In addition to these well-established techniques, another analytical approach based on the beam spread function (BSF) has been proposed (Yona et al., 2016). For validation of our light simulation, we also determined the light distribution predicted by BSF for same probe geometry and tissue parameters. The BSF simulations were done in MATLAB 2018a (The MathWorks, Natick, MA, United States) using the ScatterBrain (v0.8) software. The results of the validations are included in the Supplementary Material (Supplementary Figures 6, 7). Shortly, while the estimations obtained with the KM and BSF models agree well to each other and to existing experimental data, the MC simulations clearly overestimated the irradiance levels close to the light probe since there is almost zero scattering and absorption in the initial ballistic regime of photon propagation (for gray matter ∼140 μm) (Ntziachristos, 2010). For this reason, we chose the KM model for the rest of the study, unless indicated otherwise.

#### Optothermal Effect

Light absorption by tissue generates heat. The amount of absorbed energy is defined by the source term, which is the product of the light irradiance ϕ(mW/mm^2^) and the absorption coefficient μ_*a*_. Here, we describe the resulting temperature increase by the well-established Pennes *bioheat equation* (Pennes, 1948), which we simplify by ignoring the convection term due to blood/fluid perfusion:

(12) $$\rho \,c\,\frac{\partial T}{\partial t}=\nabla \left(k\,\nabla T\right)+\phi \,\mu_{a}.$$

Here, the constant ρ denotes the mass density (ρ = 1.04 × 10^3^ kg/m^3^), c the specific heat capacitance (*c* = 3.6 J/gK) and k the thermal conductivity (*k* = 0.56 W/m K) of the brain tissue. The forward finite difference method is used to solve Eq. 12, using a spatial step width of Δx = 0.03 mm, and ensuring numerical stability by adjusting the temporal step as given in Stujenske et al. (2015) to

(13) $$\Delta \,t\leq \frac{{\left(\Delta \,x\right)}^{2}}{6\,k}\,\rho \,c.$$

To estimate the heating effect, we used the light irradiance estimates given by the Monte Carlo simulations, as they were higher than the values given by the KM model and were therefore selected as worst-case scenario.

## Results

### Validation of the Modelled Cell Dynamics

**FIGURE 3:**
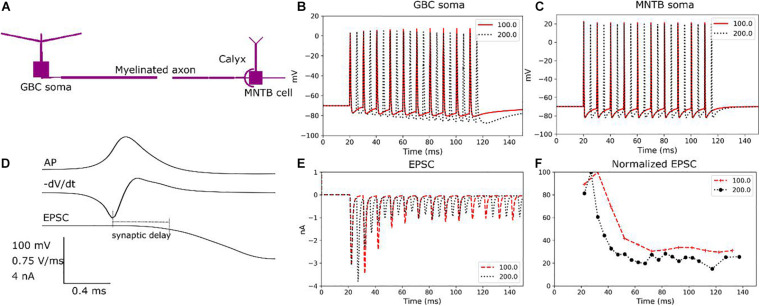
Validation of the modelled cell dynamics. **(A)** Morphologically simplified model of a GB cell connected to a principal cell. **(B)** GB cell response to current injections (0.25 ms, 20 nA) for two different stimulating frequencies (100 and 200 Hz) at the soma. **(C)** MNTB principal cell response to current injections (0.25 ms, 0.5 nA) for two different stimulating frequency (100 and 200 Hz) at the soma. **(D)** Synaptic transmission from the calyx of Held to the soma of the MNTB principal cell. An action potential and its negative time derivative are shown together with the resulting EPSC at the soma of the principal cell to indicate the synaptic delay. The soma of the MNTB principal cell was clamped to –70 mV to evoke the AP. **(E)** Trains of current pulses with frequencies of 100 and 200 Hz, respectively, are applied to the soma of the GB cell and the resulting EPSCs are determined at the soma of the principal cell. Both frequencies reliably generate APs at the calyx. **(F)** EPSCs normalized to the peak EPSC show initial facilitation followed by depression.

We confirmed that the model cells exhibited the expected dynamics using morphologically simplified models of a globular bushy cell connected to a principal cell (Figure 3A). First, we tested the responses of each individual cell to repetitive current pulses applied to the soma region. Consistent with electrophysiological data (Manis and Campagnola, 2018), the GB cell generates reliable firing for frequencies up to 200 Hz (Figure 3B). Similar results are obtained for the principal cell of the MNTB region (Brew and Forsythe, 1995; Macica et al., 2003; Figure 3C).

In order to verify the modelled synaptic transmission at the calyx, Figure 3D illustrates the synaptic currents under a voltage clamp with a holding level of −70 mV applied to the soma of the principal cell. The synaptic delay has a good match with experimental data obtained in rat brain slices for postnatal days 14 to 17 (Fedchyshyn and Wang, 2007). In addition, trains of current pulses with frequencies of 100 and 200 Hz, respectively, were applied to the GBC soma and the EPSCs were measured at the soma of the principal cell. Both cases generated highly reliable trains of APs at the calyx (Figure 3E). Since the calyx has 500 active transmission zones, it causes post-synaptic EPSCs larger than 1 nA. These EPSCs are strong enough to guarantee a suprathreshold EPSP for each pre-synaptic AP (Forsythe, 1994). For the chosen calcium concentration, the EPSC trains show an initial facilitation followed by a depression response (Figure 3F), in line with experimental data (Barnes-Davies and Forsythe, 1995). Continuing depression and facilitation, respectively, of the EPSCs could be observed when changing the extracellular calcium concentration correspondingly (data not shown) (Graham et al., 2001). In summary, the transmission model adequately represents paired-pulse facilitation, depression and AMPA receptor desensitization, as demonstrated in Barnes-Davies and Forsythe (1995).

### Feasibility of Light Excitation of a Brainstem Slice

**FIGURE 4:**
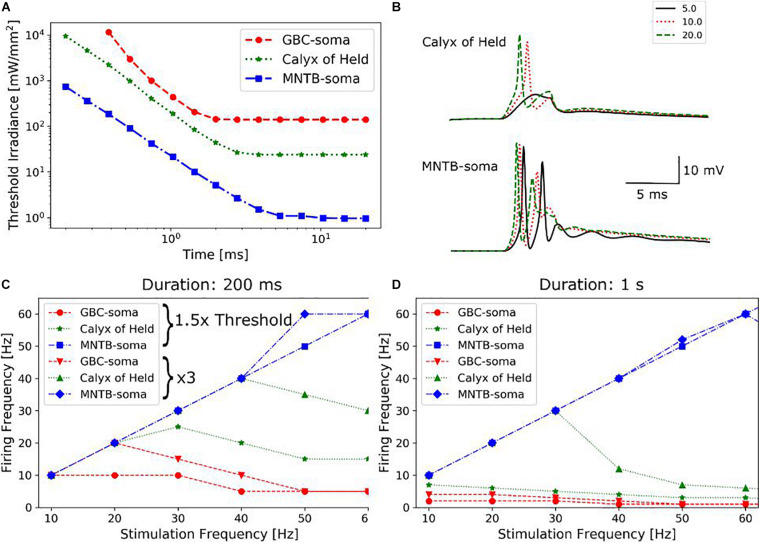
**(A)** Dependencies of the irradiance threshold on the duration of the light pulse for three different parts (soma of GB cells, Calyx of Held, soma of MNTB principal cells) of the pathway. **(B)** Illumination of the calyx of Held activates light sensitive channels of the MNTB principal cells and causes firing independent of synaptic transmission. Since the irradiance threshold of MNTB cells is much lower, they already spike under far weaker illumination. **(C)** Firing frequencies in response to repetitive light stimulation (200 ms total duration, 3 ms pulses) for the three parts of the pathway. Repetition frequencies of the light pulses range from 10 to 60 Hz, and two different irradiance levels were tested. **(D)** Total stimulation duration is increased to 1 s, with the other parameters held identical to **(C)**.

To study the feasibility of light stimulation as an alternative to the electrical stimulation, we systematically varied the intensity and duration of the light pulses to determine the irradiance threshold required to generate an action potential for three different parts of the pathway (as can be seen in Figure 2C), namely the soma of the GB cells, the calyx of Held and the soma of the MNTB principal cells (Figure 4A). The membrane potential at the 7th node of Ranvier, counted from the initial segment, was used to determine whether a cell fired or not. The irradiance threshold shows the typical dependence on pulse duration, as also commonly observed for electrical stimulation. The rheobase irradiance levels are ∼140, ∼24, and ∼1 mW/mm^2^ for the GBC soma, the calyx and the MNTB principal cell soma, respectively. The corresponding chronaxies are ∼1.3, ∼2.0, and ∼3.5 ms. The calyx is more easily excitable than the GBC soma since it can accommodate more light excited currents due to its large surface area and higher calcium channel density. However, illumination of the calyx causes also independent firing of the MNTB principal cells (Figure 4B), as they have even lower thresholds. We also tested light stimulation of the myelinated GBC axons. However, action potentials could not be triggered even at very high (>10 W/mm^2^) illumination intensities. As the nodes of Ranvier have small surface areas, even fully saturated ChR2 currents are not enough to sufficiently depolarize the membrane.

**FIGURE 5:**
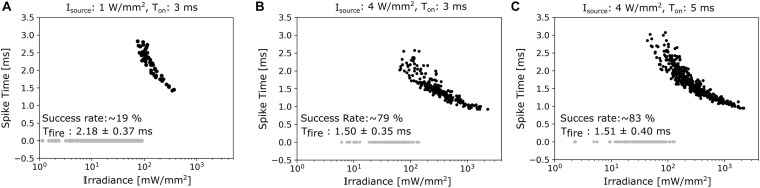
**(A)** Spike times of the 300 cells in the brainstem slice under an illumination with an irradiance level of 1 W/mm^2^ and 3 ms duration. Please see the main text for details on the light probe. Inactive cells are plotted as gray dots, activated as black dots. **(B)** The irradiance level at the source is increased to 4 W/mm^2^, causing a clear increase in the activation rate. **(C)** The duration of the stimulus pulse is increased to 5 ms for an irradiance of 4 W/mm^2^, still giving similar results as seen for the shorter pulse.

As long as magnetometer sensitivities are limited, it might be necessary to average the neural responses to many successive pulses in order to recover a sufficient SNR level. We therefore determined the firing frequencies of the modelled neurons in response to repetitive light stimuli, with varying the repetition frequencies from 10 to 100 Hz. The total duration of the pulse train was 200 ms, a single pulse had a duration of 3 ms, and illumination strength was chosen to be 1.5 and 3 times of the irradiance threshold (Figure 4C). The responses became quickly unstable for the lower illumination strength when increasing stimulation frequency. At the higher illumination strength, the responses became unstable for frequencies exceeding 15 Hz when stimulating the GBC soma. In contrast, stimulation of the calyx gave stable responses for frequencies up to 40 Hz. When increasing the duration of the pulse train to 1 s (Figure 4D), reliable stimulation of the GBC soma was not possible, while calyx stimulation was still stable up to a frequency of 30 Hz. The soma of the MNTB cells could accommodate even higher frequencies at both pulse train durations. However, as this activation does not result in action potentials traveling along the GBC axon pathway, frequencies exceeding 40 Hz might be of less practical relevance. On the other hand, varying between low and high frequency stimulation might give the possibility to differentiate between the relative contributions of the calyx and the soma of the principal cells to neural recordings. In general, these results suggest that optogenetic stimulation requires the use of lower stimulation frequencies than electric stimulation, as the slow closing mechanism of ChR2 channels causes a longer spiking latency (Grossman et al., 2011a). When firing in synchrony, the neural magnetic fields of the stimulated cells add up, increasing the signal recorded by the magnetometer. As this effect depends on the exact temporal alignment of the single fields, we investigated the timing of action potentials generated by 300 GB cells distributed in a 300 μm thick slice under three different lighting conditions (1 W/mm^2^ light source irradiance and 3 ms duration; 4 W/mm^2^ and 3 ms; 4 W/mm^2^ and 5 ms). The light distribution in the tissue was simulated with the KM model. We selected a light irradiance at the source of 1 mW/mm^2^ as this is at the upper limit for the available optogenetic solutions using “fiber-coupled” LED light sources, which can output a power of up to 30 mW (Prizmatix, 2019). This irradiance was increased four-fold to 4 mW/mm^2^ as a test for an extreme case. The light probe (optical fiber) was simulated 25 μm away from the slice and centered above the MNTB area to excite the calyces. The diameter was set to 200 μm and the NA to 0.37. For stimulation with 1 W/mm^2^ and 3 ms, 19% of the GB cells were antidromically activated in the complete slice. Mean firing times were 2.18 ms (±0.37 ms standard deviation, SD). Increasing the light irradiance at the source to 4 W/mm^2^ (still at 3 ms duration) resulted in an activation of 79% of the cells with mean firing times of 1.50 ± 0.35 ms SD (Figure 5B). The spatial distribution of activated and inactivated neurons using different light stimulation methods can be seen in Supplementary Figure 9. Using longer pulses of 5 ms gave similar results (Figure 5C). These results indicate that optical stimulation is limited in its ability to activate all cells in high synchronicity, which will result in weaker and temporally more dispersed signals recorded by the magnetometer when compared to the signals caused by electrical stimulation.

### Spatial-Temporal Characteristics of the Neural Magnetic Activity of a Brainstem Slice

**FIGURE 6:**
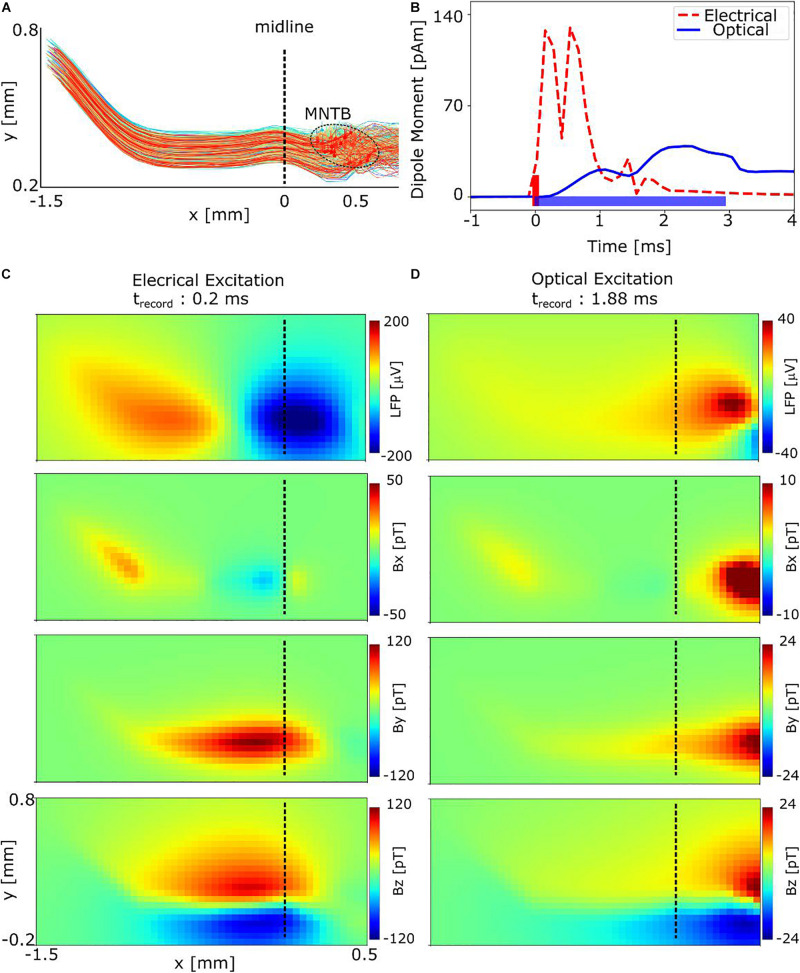
Simulation results showing the spatial distributions of the extracellular electric potential and neural magnetic fields of the auditory GBC-MNTB pathway in response to electrical and optical stimulation of the MNTB region. **(A)** The simulated pathway consists of 300 GB cells distributed in a 300 μm thick slice, connected to 300 MNTB principal cells. The stimulated MNTB region is indicated by the dashed ellipse. **(B)** Equivalent current dipole moment. **(C)** LFP and magnetic fields at *t* = 0.2 ms after the start of the electrical pulse. The field distributions are in accordance with an axial current flow from the axonal pathway to the MNTB region, caused by action potentials traveling antidromically along the pathway. **(D)** Magnetic fields at *t* = 1.88 ms after start of the light pulse. The spatial distributions around the MNTB region differ from those caused by electrical stimulation. Having lower light excitation thresholds, more MNTB principal cells than GB cells are activated, which makes the contribution of the former more dominant (see Supplementary Figure 3A). Still, the overall lower number of activated cells and the temporal dispersion of the activations decreases the peak field strength by a factor of approx. five.

Our main interest was the estimation of the spatial-temporal characteristics of the neural magnetic fields that are caused by the stimulation of the auditory pathway in a transverse brainstem slice. An active cell layer thickness of 300 μm (Figure 6A) and a temperature of 34°C was chosen for the simulations. The distance from the diamond sensor to the active cell layer was set to 25 μm to account for the layer of inactive cells due to the cutting procedure. The magnetic field and the electric potential caused by the neural activity was calculated at a surface area with a size of 2 × 1 mm^2^. The surface area was discretized into a 50 × 25 grid, resulting in an in-plane resolution of 40 μm. Considering a conduction velocity of 5 to 10 m/s and a depolarization time of 0.1 ms, such spatial sampling is considered sufficiently high to accurately capture the spatial features of the magnetic fields.

Given that the calyces of Held and the soma of the principal cells had lowest irradiance thresholds, we simulated the electrical and optical stimulation of the MNTB region in the following. Our aim was to characterize the contributions of the neural activity at the different parts of the pathway to the overall magnetic fields that can be recorded at the surface of the brain slice. In the electrical stimulation case, the stimulus was chosen to have a duration of 0.1 ms. As idealized reference case, we used intracellular current injections that were placed at the calyces of Held and their amplitudes set to 5 nA, which is strong enough to generate synchronous events at all calyces of Held. In addition, to evaluate the feasibility of the generation of synchronous events by extracellular stimulation, a monopolar electrode was simulated at a depth of 200 μm of the slice and centered at the MNTB area to excite the calyces (details on the implementation can be found in Supplementary Section 6). In the optical stimulation case, a light probe with an NA of 0.37 and a diameter of 0.2 mm was simulated on the top of the slice at a distance of 25 μm. The source irradiance at the probe was set to 1 W/mm^2^ (∼30 mW light power) and the stimulus had a duration of 3 ms.

Since neural magnetic fields strongly depend on the neural axial current densities (Swinney and Wikswo, 1980; Karadas et al., 2018), we also determined an equivalent current dipole (ECD) by summing the resulting neural currents (Figure 6B). The ECD is given by Q→⁢∑kIik⁢L→k, with ${\vec{L}}^{k}$ indicating the length of the k^*th*^ cylindrical compartment and $I_{i}^{k}$ the axial current flow within the compartment at the time frame i. The simulated ECD reached its peak strengths 0.20, 0.15, and 1.88 ms after start of the intracellular electrical, extracellular electrical and optical stimulation, respectively. Therefore, we determined the spatial distribution of the neural magnetic fields and local field potentials (LFPs) at these time points. The results for intracellular electrical stimulation are shown in Figure 6C. At the chosen time point, the GB cells are depolarized, and a strong axial current flow is started from the myelinated pathway at the left side of the midline to the calyces of Held. As a result, the LFP has a dipole distribution centered around the midline of the brain slice. Determined by the orientation of the axon pathway at this position, the neural source currents are predominantly oriented in x direction. The spatial pattern of the resulting magnetic field is in accordance to this direction, with the Bx component being almost zero. The magnitude of the By and Bz components reach peak values up to 120 pT at the distance of 25 μm for the worst-case estimate of 300 cells in the slice. Considering a more likely estimate of 1,250 cells in the slice, the peak signal reaches ∼500 pT (results obtained by linear rescaling of the field of the 300 cells). In general, axial currents in the myelinated axon pathway contribute most to the magnetic fields. For example, selecting a later time point at which only the soma of the MNTB principal cells are activated due to synaptic transmission (delay: ∼0.6–0.7 ms) results in almost four times lower peak fields (Supplementary Figure 4A). Likewise, the antidromic activation of the soma of the GB cells causes only weak fields (Supplementary Figure 3).

For extracellular rectangular stimuli with 100 μA and 100 μs, 82% of the GB cells are antidromically activated in the complete slice. Considering a titanium nitrite (TiN) electrode with 10 μm diameter, this level of current strength and duration is below the safe-charge injection limits to avoid damage to the electrode and cells (Multi Channels Systems MCS GmbH, 2018). Changing from intra- to extracellular stimulation only weakly affects the synchrony of the generated APs. For extracellular stimulation, the APs were mostly generated by activation of the 1st or 2nd node (counted from the calyx). The firing times of the (arbitrarily selected) the 6th node of the axon, again counted from the calyx, were 0.09 ms with a low standard deviation (SD) of ± 0.04 ms after stimulus onset, demonstrating the good synchrony of the APs. As a result, the magnetic peak fields seem to be only moderately reduced to ∼100 pT for our worst-case estimate of 300 cells in the slice (Supplementary Figure 12). Similar magnetic peak fields are obtained when an electrode is placed in the VCN region close to the soma of the GB cells, where ∼80% of the cells are activated with the same stimulation parameters via triggering APs at the ends of the axon initial segments (data not shown). Activation of 100% of the cells could be obtained when increasing the stimulus level to 200 μA, which, however, might result in damage of the electrode tip or the surrounding cells (Multi Channels Systems MCS GmbH, 2018) (data not shown).

In the optical stimulation case, the peak strengths of the fields decrease approximately fivefold (Figure 6D), in accordance to the weaker and temporally broadened ECD. This effect results from the temporal asynchrony of the generated action potentials and low number of excited cells (Figure 5A), which also causes a broadening of the LFP and the magnetic field of GB cells along x direction (i.e., along the main direction of the axon pathway; Supplementary Figure 4B). In addition, the magnitudes of the fields at the MNTB region are higher than those at the axon pathway and dominate the overall magnetic field in case of optical stimulation (Figure 6B). This is because the contribution of MNTB cells to the recorded magnetic fields is almost two times higher than that of the GB cells (Supplementary Figure 4B) due to their lower excitation thresholds.

### Effect of Calyx Morphology

We aimed to ensure that the rather weak magnetic fields observed at early time points after stimulation were not biased by the chosen morphological models for the calyces of Held which were represented as half rings. Therefore, we tested three different variants for these models with increasing morphological complexity (Supplementary Figure 6A). The second variant modelled the calyx as an additional half ring perpendicular to the first one with two simple sticks of lengths of 6 μm, placed at random orientations. The third variant successively added additional sub-branches to reach better approximations of the real calyx morphology. However, the resulting magnetic field was only weakly affected by the model complexity (Supplementary Figures 6E,F).

### Heating Effect of Light Excitation

We wanted to ensure that tissue heating due to optical absorption does not pose a problem when applying many light pulses at high repetition rates in order to be able to obtain averaged data with sufficient SNR. We thus estimated the light distribution in brain tissue and used this as input to the bioheat equation to estimate the temperature change. In order to obtain robust worst-case estimates, we used Monte Carlo simulations here as they predict higher irradiance levels than the KM model (see Supplementary Figures 7, 8). Two different numerical apertures (NA = 0.58 and NA = 0.37) and two probe diameters (200 and 500 μm) were tested to assess the impact of these parameters on the resulting light distribution. As NA had only little influence, it was kept fixed at 0.37 when evaluating the resulting heating.

**FIGURE 7:**
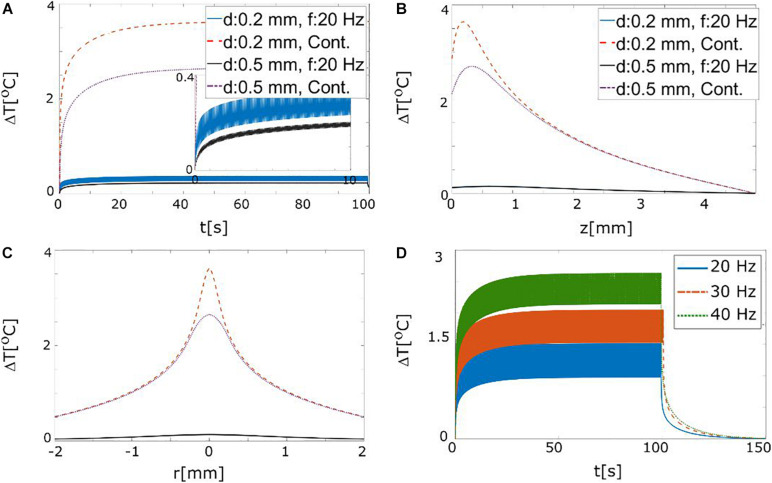
**(A)** Maximal temperature changes in the tissue (*z* = 200 μm) in response to continuous and pulsed (3 ms duration and 20 Hz) stimulation with 30 mW output power for probes with 200 and 500 μm diameter (NA = 0.37). **(B)** Spatial profile of the temperature increase (*t* = 10 s) along the axial (z) direction. **(C)** Spatial profile of the temperature increases along radial direction for *z* = 200 μm. **(D)** Maximal temperature changes for different stimulation frequencies (source irradiance 4 W/mm^2^, pulse duration 3 ms). An irradiance level of 4 W/mm^2^ at the light probe corresponds to a light power of ∼120 mW from an optical fiber with a diameter of 200 μm. The temperature increases proportionally with the frequency.

We tested continuous and pulsed (3 ms pulse duration and 20 Hz repetition rate) stimulation with a light source irradiance of 1 W/mm^2^ that illuminated a cubic tissue volume of 1 mm^3^. The initial temperature increase saturates after ∼10 s (Figure 7A), where it reaches 2.5–3.5°C for continuous stimulation (Figures 7A–C). For the pulsed stimulation, the effective source light irradiance is decreased to ∼64 mW/mm^2^ and therefore also the heating effect is reduced by an order of magnitude. Maximal temperature changes for different stimulation frequencies were also tested (Figure 7D). For an irradiance level of 4 W/mm^2^ (corresponding to a light output power of ∼120 mW), a temperature rise of 2°C is expected for 40 Hz stimulation. Considering that these choices of the stimulation frequency and maximal output power represent a worst-case scenario (see Figures 4, 5 and the corresponding results sections), we can safely conclude that the temperature rise will stay below 2°C. Very high light intensities, such as the ones used in this model, may cause other types of photochemical damage, however, such effects were beyond the scope of the present study.

## Discussion

The main aim of this study was to evaluate the spatiotemporal characteristics of the neural magnetic fields of the auditory VCN-MNTB pathway in transverse slices of the mouse brainstem. For this purpose, we constructed a network model with realistic dynamics and morphology and simulated the magnetic fields in response to electrical and optical stimulations. Our results suggest that the peak magnetic field strengths of ∼0.5 and ∼0.1 nT should be expected from a 300 μm thick brainstem slice, after electrical and optical stimulations, respectively, when assuming that ∼1,250 MNTB cells are contained in the slice. That is, while optical stimulation would be beneficial to avoid artefacts in the signal that occur for electric stimulation, it also results in substantially weaker neural magnetic fields. Increasing the neural response to optical stimulation would likely require a higher light power than achievable with currently available commercial systems (>100 mW) to activate a sufficient amount of the cells at the same time, then reaching peak magnetic fields of ∼0.05 nT for 300 MNTB cells (Supplementary Figure 11), or ∼0.2 nT for the more optimistic assumption of 1,250 cells. While a light power of 100 mW can currently not be realized, it is worth noting that the temperature changes would still be limited to a physiologically acceptable range given that a pulsed stimulation mode is employed.

Characterizing the neural magnetic response to optical stimulation was one of the main aims of this simulation study. In the following, we will therefore discuss methodological factors that may affect the reliability of simulating optical stimulation. In addition, we give an overview of putative improvements of the efficacy of optogenetic stimulation. We will then relate the simulated neural response to the sensitivity levels that have been achieved for NV-based magnetic field recordings to give an estimate of the practical feasibility of the suggested recording approach. We conclude with a brief summary and outlook.

### Simulating and Improving the Neural Magnetic Response to Optical Stimulation

Our estimations of the extracellular neural fields that can be triggered by optical stimulation were based on methods that rely in part on simplifying assumptions and uncertain parameters. We tested three different methods to estimate the light distribution in the brain slice. The Kubelka-Munk (KM) model is widely used in optogenetics, even though it relies on the simplifying assumption of isotropic scattering. In order to test for the impact of tissue anisotropy on the results, we therefore also performed simulations with a MC method (Liu et al., 2015). Finally, we tested a beam spread function (BSF) method that was proposed in a recent study to overcome the limitations of the KM model and especially to estimate the optical parameters from the measured attenuation curves (Yona et al., 2016). Generally, we found a good agreement between the results of the KM model and the BSF method, which both predict similar numbers of neuronal activations for the simulated stimulation protocols (Supplementary Figure 9). Their results were also in agreement with published experimental data on the light distribution and attenuation in brain gray matter (Aravanis et al., 2007; Gradinaru et al., 2009; Yizhar et al., 2011; Al-Juboori et al., 2013; Supplementary Figures 7, 8). We therefore utilized the KM model to study the cell responses to optical stimulation. However, our MC simulations gave deviating results, as apparent from a clear underestimation of the attenuation by the brain tissue (Supplementary Figure 7). The reasons for this discrepancy is likely that the MC does account for attenuations in the ballistic regime of photon propagation, which was our region of interest. Still, we decided to use the MC simulations to obtain robust worst-case estimates of the tissue heating.

Another important aspect of the modeling of optical stimulation was the selection of the density of the photo-sensitive channels in the neural membrane, as this has a direct influence on the irradiance threshold and illumination volume of the tissue (Foutz et al., 2012). Its absolute value is unknown and, in line with common practice (Grossman et al., 2011a), we assumed a constant ChR2 density of 1.3e10 channels/cm^2^, based on results for the bacteriorhodopsin expression in an oocyte (Nagel et al., 1995). However, a higher density value was estimated (4.41e12 channels/cm^2^) from the experimental data of the mammalian nervous system (Arlow et al., 2013). When using this very high channel density, the strength of the equivalent current dipole of the optically triggered neural activity increases by a factor of three (Supplementary Figure 10), but only for the case of very high source irradiance (3.2 > W/mm^2^, corresponding to an optical power of ∼100 mW). Therefore, we think that our finding of strongly reduced peak fields for optical versus electric stimulation holds robustly.

In a related manner, it was interesting to see that merely increasing the light power was not sufficient to achieve highly synchronous activations of all neurons in the brain slice, and that the maximally achievable repetition rate was lower for optical versus electric stimulation. These limitations were also caused by the comparatively slow dynamics and the low conductance of the light-sensitive ChR2 channels, which lowered the strength of the optically controlled membrane currents. Increasing channel density by increasing ChR2 expression might help to ameliorate these problems, but this approach can also change the native membrane environment (Lin, 2010). An alternative and favored approach is increasing the conductance and changing the channel kinetics by developing a ChR2 mutant to achieve (i) a lower threshold to light stimulation, (ii) a better efficiency at high frequencies, and (iii) red shifting the spectral response to reach deeper cells with minimal light scattering and absorption (Lin, 2010). With this aim, the E123T (ChETA) (Gunaydin et al., 2010) and ChD mutations (Lin et al., 2009) were developed for faster opening/closing rates. Channels bearing the abovementioned mutations achieve improved spiking efficiency at high frequencies (up to 200 Hz) at the cost of higher light thresholds (Grossman et al., 2011a). Another mutant (ChIEF) was developed to provide better light sensitivity with more stable photocurrent responses during continuous light or high frequency pulsed light (Lin et al., 2009; Lin, 2010). Therefore, choosing ChETA or ChIEF instead of ChR2 might help to increase synchrony of spiking or to reach higher spiking frequencies. However, we expect the achievable increases of the strength of the extracellular magnetic fields to be only modest, because the generation of synchronous APs throughout the slice would still require a light power which is currently not feasible in practice.

Recently, the expected temperature rise and its effect on the tissues during optical stimulation has been studied in rodent brain *in vivo* (Stujenske et al., 2015; Senova et al., 2017). These studies conducted optical stimulation of cortical regions in rats and found temperature rises in the range of 0.1–2.5°C, depending on the fiber and stimulation parameters (Senova et al., 2017). The authors did not report any change in neural activity due to heating for stimulation with up to 200 mW/mm^2^ continuous power. Considering low frequency (∼30 Hz) pulsed stimulation, our simulation indicate temperature increases in a similar range with a light intensity of up to 4 W/mm^2^ at the fiber tip (Figure 7D). This suggests that increasing light power to get higher activation should not be considered as a main concern for pulsed optical stimulation.

### Relating the Neural Magnetic Fields to the Sensitivity of NV-Based Magnetic Field Recordings

In this section we discuss how the simulated magnetic fields can be detected by the use of current setups of NV diamond sensors. The aim was to predict the signal from an experimental setup similar to the one described in Webb et al. (2021). However, the modeled magnetic fields are independent on the sensor setup. In an experimental NV sensor setup, as seen in e.g., (Webb et al., 2021), the diamond sensor is illuminated from below with a laser with a wavelength of 532 nm (green), and the resulting fluoresce light at ∼650 nm (red) is measured which has an intensity that is directly proportional to the magnetic field. The light is collected through a 600 nm long pass filter to remove any potential interference from the blue light used for ontogenetic stimulation. In principal, the blue light could excite the NV centers. However, they have very low absorption at this wavelength, and the effect would be negligible. Furthermore, in Webb et al. (2021) the light was shielded from the brain slice with a layer of reflective aluminum, so that the interferences between the two different optical setups (i.e., optogenetic stimulation from above, and NV diamond sensor excitation from below) become negligible.

The sensitivity of diamond sensors are limited by the density of the NV ensemble in the diamond, the dephasing time $T_{2}^{*}$ and the on-resonance fluorescence contrast (Balasubramanian et al., 2008; Taylor et al., 2008). These parameters are determined by factors such as the isotopic composition of the diamond (Balasubramanian et al., 2009), material strain (Kehayias et al., 2019) and the growth method used (Acosta et al., 2009). For neural signals in the DC to low kHz range (millisecond timescale), continuous wave protocols are so far employed for sensing which have achieved volume-normalized sensitivities of up to $\eta_{V}\,34\,n\,T\,m^{\frac{3}{2}}\,H\,z^{-\frac{1}{2}}$ using an isotopically engineered diamond with *n*_*NV*_∼1ppm and ${\text{T}}_{2}^{*}∼0.5$ μs for sensing from a biological sample (Barry et al., 2016). When assuming a sampling rate of 10 kHz to reliably reconstruct the shape of the APs and a 5 μm-thick layer of NV centers in the diamond sample, this volume-normalized sensitivity corresponds to an area-normalized sensitivity of around 1,520 nT μm (applying Karadas et al., 2018, Eq. 9). Reasonable reconstructions of the 2D spatial distribution of neural magnetic fields in the range of 0.5 nT require area-normalized sensitivities of 10 nT μm or better (Karadas et al., 2018), so that several thousands of trials would need to be averaged to achieve sufficient reconstruction quality when using existing technology. However, sensitivity improvements of more than two orders of magnitude have been discussed for the next generation NV sensors (Barry et al., 2020), which would render the NV-based imaging of neural magnetic fields feasible.

Although sensitivities in the sub-pT/Hz^1/2^ range have recently been reported for NV-based magnetic field sensing (Fescenko et al., 2020; Zhang et al., 2021), measurements of biological samples so far only reached picotesla ranges. This includes 15 pT/Hz^1/2^ sensitivity for measurements on an invertebrate sample (Barry et al., 2016) and 50 pT/Hz^1/2^ for mouse tissue (Webb et al., 2021), with a noise level of 12 pT being reached after 8 h of optical stimulation at 0.5 Hz. These sensitivities are already sufficient to measure the expected signals in the range of 0.5 nT and lower. They were reached when sensing the average magnetic field in the complete measurement area of the diamond (mm-scale) – essentially measuring the average of the magnetic fields as seen in Figure 6 – and thus sacrificing information about the spatial distribution of the fields. This is a promising intermediate step toward further maturing this novel technology. Obviously, the amplitude of the averaged signal depends on the sensor position relative to the sample. Even for close-by positions, the signal may be close to zero when the signal from areas with field in- and decreases are spatially averaged. This can be most clearly be seen for the Bz-field component in Figure 6 for the present model. In this respect, simulations of the expected spatial field distribution can be helpful to avoid such cancellation effects. Another possible method of getting rid of these cancellation effects and increasing the spatial resolution could be achieved by decreasing the sensor area, for example by using an NV diamond scanning probe, as done by Thiel et al. (2019).

## Conclusion

In conclusion, our results serve as a detailed characterization of the electric and magnetic neural signals emitted from the auditory brainstem pathway of a mouse brain in response to optic or electric stimulation. Our results suggest that the APs traveling along the axonal pathway from GB cells to MNTB principal cells will dominate the imaged neural magnetic fields, while magnetic signals from the calyces of Held or the MNTB principal cells will be far weaker. The aim was to evaluate the feasibility of measuring the magnetic field from this neuronal pathway using existing NV diamond sensor technology, and the results showed that this pathway did not elicit sufficiently strong signals for spatially resolving the signals in these structures with either stimulation method. In contrast, the magnetic fields of pyramidal cells in hippocampal brain slices yield much stronger magnetic fields (Karadas et al., 2018) as they are stronger and have slower dynamics (thus allowing for lower sampling rates), which both will increase the SNR of the measurements. While avoiding artefacts in the recorded signals, the optical stimulation method consistently produced much lower peak magnetic fields (0.1 nT) than the electrical stimulation method (0.5 nT), even when the optical stimulation light irradiance at the source was increased to 4 W/mm^2^, which exceeds what is practically reasonable.

Although NV sensor technology is not currently able to image neural magnetic signals to allow distinguishing between different neural sources along the investigated pathway, as is currently done using e.g., fluorescent imaging, the present results suggest that detecting the neural magnetic fields by spatial averaging the field over a whole mm-sized diamond (as done by Barry et al., 2016; Webb et al., 2021) would likely be possible. In order to reach maximal field strengths, the presented model and stimulation results are highly useful for determining good sample positioning for such methods. The simulation approach of neural magnetic fields in response to optical stimulation, as presented here, is flexible and can be easily extended to other brain regions that differ in light scattering and absorption properties. We also expect our approach to be useful to simulate the excitation of various neuronal types under different configurations of light excitation, and to optimize light distribution and illumination conditions.

## Data Availability Statement

The raw data supporting the conclusions of this article will be made available by the authors, without undue reservation.

## Author Contributions

MK contributed to the study design, performed the simulations, analyzed and interpreted the data, and drafted a first version of the manuscript. CO contributed to the analysis and interpretation of the data, wrote sections of the manuscript, and led the manuscript revisions. AT contributed to the study design, the interpretation of the data, and the drafting of the first version of the manuscript. NW, J-FP, AH, and UA contributed to the study design. JW wrote a section of the manuscript. All authors contributed to manuscript revision, read, and approved the submitted version.

## Conflict of Interest

The authors declare that the research was conducted in the absence of any commercial or financial relationships that could be construed as a potential conflict of interest.
