# Supplementary material for: In-vitro Recordings of Neural Magnetic Activity From the Auditory Brainstem Using Color Centers in Diamond: A Simulation Study
Source: Front Neurosci. 2021 May 13;15:643614. doi: 10.3389/fnins.2021.643614 (PMC8155532; doi:10.3389/fnins.2021.643614)
Supplement: Supplementary file 1 [file Data_Sheet_1.pdf]

Supplementary Material

Channel Kinetics of the GBC-MNTB Pathway

S.1 Ion Channel Model of the GB cells

The equations and parameters used to describe the channel kinetics were taken from (Rothman and Manis, 2003; Ford et al., 2015). The ionic currents are governed by an activation/inactivation variable s whose rate of change is defined by

$$\frac{ds}{dt} = \left(\frac{\alpha_s}{\alpha_s + \beta_s} - s\right)(\alpha_s + \beta_s) \tag{1}$$

Sodium Current:

$$I_{Na} = \bar{g}_{Na} m^3 h (V - E_{Na}) \tag{2}$$

$$\alpha_m = \frac{0.36(V + 49)}{1 - \exp\left[-\frac{V + 49}{3}\right]} T_f(3,22) \tag{3}$$

$$\beta_m = \frac{-0.4(V + 58)}{1 - \exp\left[-\frac{(V + 58)}{20}\right]} T_f(3,22) \tag{4}$$

$$\alpha_h = \frac{2.4}{1 + \exp\left[\frac{V + 68}{3}\right]} T_f(3,22) + \frac{2.4}{1 + \exp[V + 61.3]} T_f(10,22 \tag{5}$$

$$\beta_h = \frac{3.6}{1 + \exp\left[-\frac{V + 21}{10}\right]} T_f(3,22) \tag{6}$$

Low – Threshold K^+ current:

$$I_{K_{LT}} = \bar{g}_{K_{LT}} w^4 z (V - E_{K_{LT}}) \tag{7}$$

$$\alpha_w = \frac{\left(1 + \exp\left(-\frac{V+48}{6}\right)\right)^{-\frac{1}{4}}}{100\left(6\exp\left(\frac{V+60}{6}\right) + 16\exp\left(-\frac{V+60}{45}\right)\right)^{-1} + 1.5} T_f(3,22) \tag{8}$$

$$\beta_w = \frac{\left(1 - \left(1 + \exp\left(-\frac{V+48}{6}\right)\right)^{-\frac{1}{4}}\right)}{100\left(6\exp\left(\frac{V+60}{6}\right) + 16\exp\left(-\frac{V+60}{45}\right)\right)^{-1} + 1.5} T_f(3,22) \tag{9}$$

$$\alpha_z = \frac{1 + \left(1 + \exp\left(-\frac{V+71}{10}\right)\right)^{-1}}{2000\left(\exp\left(\frac{V+60}{20}\right) + \exp\left(-\frac{V+60}{8}\right)\right)^{-1} + 100} T_f(3,22) \tag{10}$$

$$\beta_h = \frac{1 - \left(1 + \exp\left(-\frac{V+71}{10}\right)\right)^{-1}}{2000\left(\exp\left(\frac{V+60}{20}\right) + \exp\left(-\frac{V+60}{8}\right)\right)^{-1} + 100} T_f(3,22) \tag{11}$$

High – Threshold K⁺ current:

$$I_{K_{HT}} = \bar{g}_{K_{HT}}(0.85n^2 + 0.15p)\left(V - E_{K_{HT}}\right) \tag{12}$$

$$\alpha_n = \frac{\left(1 + \exp\left(-\frac{V+15}{5}\right)\right)^{-\frac{1}{2}}}{100\left(11\exp\left(\frac{V+60}{24}\right) + 21\exp\left(-\frac{V+60}{23}\right)\right)^{-1} + 0.7} T_f(3,22) \tag{13}$$

$$\beta_n = \frac{\left(1 - \left(1 + \exp\left(-\frac{V+15}{5}\right)\right)^{-\frac{1}{2}}\right)}{100\left(11\exp\left(\frac{V+60}{24}\right) + 21\exp\left(-\frac{V+60}{23}\right)\right)^{-1} + 0.7} T_f(3,22) \tag{14}$$

$$\alpha_p = \frac{\left(1 + \exp\left(-\frac{V+23}{6}\right)\right)^{-1}}{100\left(4\exp\left(\frac{V+60}{32}\right) + 5\exp\left(-\frac{V+60}{22}\right)\right)^{-1} + 5} T_f(3,22) \tag{15}$$

$$\beta_p = \frac{1 - \left(1 + \exp\left(-\frac{V+23}{6}\right)\right)^{-1}}{100\left(4\exp\left(\frac{V+60}{32}\right) + 5\exp\left(-\frac{V+60}{22}\right)\right)^{-1} + 5} T_f(3,22) \tag{16}$$

Calcium current:

$$I_{Ca} = \bar{g}_{Ca}s^2(V - E_{Ca}) \tag{17}$$

$$\alpha_s = 1.78\exp\left(\frac{V}{23.3}\right) T_f(3,24) \tag{18}$$

$$\beta_s = 0.14\exp\left(-\frac{V}{15}\right) T_f(3,24) \tag{19}$$

Hyperpolarization-activated cation current:

$$I_h = \bar{g}_h r(V - E_h) \tag{20}$$

$$\alpha_r = \frac{\left(1 + \exp\left(\frac{V+76}{7}\right)\right)^{-1}}{10^5\left(237\exp\left(\frac{V+60}{12}\right) + 17\exp\left(-\frac{V+60}{14}\right)\right)^{-1} + 25} \tag{21}$$

$$\beta_r = \frac{1 - \left(1 + \exp\left(\frac{V+76}{7}\right)\right)^{-1}}{10^5\left(237\exp\left(\frac{V+60}{12}\right) + 17\exp\left(-\frac{V+60}{14}\right)\right)^{-1} + 25} \tag{22}$$

Leakage current:

$$I_L = g_L(V - E_L) \tag{23}$$

In above equations, the rate constants of the channel kinetics were originally determined for a reference temperature T_0 (in ^oC). Therefore, they are scaled by a temperature factor T_f to adjust them according to the ambient temperature ($T_{ambient} = 34°$ was used in the simulations):

$$T_f(Q_{10}, T_0) = Q_{10}^{(T_{ambient} - T_0)/10} \tag{24}$$

**Table S1: Model parameters of the GB cell**							
	Compartment						E_{rev} (mV)
Active (mS/cm^2)	Soma	Primary Dendrite	Initial segment	Node	Heminode	Calyx	
$\bar{g}_{Na}$	17.3	8.7	103.8	588	588	-	55
$\bar{g}_{K_{LT}}$	2.8	1.4	2.8	40	40	-	-90
$\bar{g}_{K_{HT}}$	2.0	1.0	4.0	-	-	20	-90
$\bar{g}_{Ca}$	-	-	-	-	-	3	43.5
$\bar{g}_h$	1.0	0.5	0.5	-	-	0.095	-43
Passive							
g_L (mS/cm^2)	0.07	0.035	0.07	1.76	-80	0.094	
E_L (mV)	-70	-70	-70	-72	-72	-90	

Note: The channel parameters at the initial segment of the axon hillock are modified to set the conductance of the hyperpolarization-activated cation channels and the Na channels to 0 and 69.2, respectively (mS/cm2).

Using the above equations, the total membrane current flow at the soma can be written as:

$$-C_m \frac{dV}{dt} = I_{Na} + I_{K_{LT}} + I_h + I_L \tag{25}$$

For Ranvier nodes, the equation for the current flow across the membrane is

$$-C_m \frac{dV}{dt} = I_{Na} + I_{K_{LT}} + I_L, \tag{26}$$

and the equation governing the current flow across the membrane in the calyx of Held is

$$-C_m \frac{dV}{dt} = I_{Ca} + I_{K_{HT}} + I_h + I_L. \tag{27}$$

S.2 Internode Model of the GBC axon

We tested two established models for myelinated axon (Model B and C of Richardson et al., 2000) (Fig. S4). The periaxonal space is not included in the model. Furthermore, it is assumed that the conductivity of the myelin membrane is constant. Therefore, the effective conductivity of the myelin sheath, R_{sheath}, for a radial current flow from the axon can determined by the series combination of all the membranes in the sheath, i.e.

$$R_{sheat} = \frac{R_{per\ membrane\ area}}{2\pi L}\sum_{i=1}^{N}\frac{1}{r_i} \approx \frac{R_{per\ membrane\ area}}{2\pi L\ d}\ \ln\left(\frac{r_{fiber}}{r_{axon}}\right) \tag{28}$$

where L is the internode length, d is the thickness of each membrane in the sheath and the r_i are the radii of the individual myelin membranes making up the sheath (assumed to occur at equally spaced radii between the axon radius and the outer radius of the sheath). This would lead to an effective trans-sheath conductivity per surface area of axon defined by

$$\begin{aligned} R_{effective} &= R_{sheat} 2\pi L\ r_{axon} \\ &= R_{per\ membrane\ area}\frac{r_{axon}}{d}\ \ln\left(\frac{r_{fiber}}{r_{axon}}\right) \end{aligned} \tag{29}$$

In electrical stimulation, the two models do not give any significant difference in terms of membrane potential changes and the external magnetic fields (Fig. S5). However, for optical simulation, we only used model B since model A cannot accommodate axonal transmembrane mechanisms for modeling light sensitive channels.

S.3 Ionic Current Model of the MNTB Principal Cells

The ionic currents were defined by (Wang and Kaczmarek, 1998)

$$I_x = \bar{g}_x\, a_x^{\lambda_x}(1 - \gamma_x + \gamma_x b_x)\big(V - V_{rev,x}\big) \tag{30}$$

where g_{max} is the maximal conductance; a is the activation rate; λ is the of subunits to activate; γ is the fraction of total conductance than can inactive; b is the inactivation rate; and V_{rev} is the reversal potential for ion x. These parameters are given in Table S2. The change in subunits a and b was given by following differential equations:

$$\frac{ds}{dt} = \left(\frac{\alpha_s}{\alpha_s + \beta_s} - s\right)(\alpha_s + \beta_s) \tag{31}$$

where s corresponds to subunit-type m, h, r, n, p or u. For each subunit the voltage dependency of the

rate constants was:

$$\alpha_s = k_\alpha e^{\eta_\alpha V} \tag{32}$$

$$\beta_s = k_\beta e^{\eta_\beta V} \tag{33}$$

The constants k_α, η_α, k_β and η_β are listed in Table S3.

The channels dynamics were adjusted for an ambient temperature of 34°C by scaling the them with Q_{10} values of 3 for Na- and low-threshold K-channels, 2.36 for Na-channel inactivation, and 5 for high-threshold K-channel activation (Lorteije et al., 2009).

The total membrane currents of the soma of the MNTB cells are then described by

$$-C_m \frac{dV}{dt} = I_{Na} + I_{LTK} + I_{HTK} + I_L + I_{syn} \tag{34}$$

where I_L is the leakage current, I_{Na} the sodium current, I_{LTK} the low-threshold potassium current and I_{HTK} is the high-threshold potassium current. I_{syn} is the synaptic current.

The total membrane currents of the dendrites, axon of the MNTB cells are described by

$$-C_m \frac{dV}{dt} = I_{Na} + I_{LTK} + I_{HTK} + I_L \tag{35}$$

The channel dynamics of the nodes of myelinated axons were chosen identical to the dynamics of the MNTB principal cell axon. The dynamics of the internodes are modeled like internodes of GBC.

Table S2: Conductance parameters for the MNTB principal cell

	I_{Na}	I_{LTK}	I_{HTK}	I_L
a	m	l	n	1
b	h	r	p	-
λ	3	1	3	1
γ	1	1	0.2	0
V_{rev} (mV)	51	-105	-105	-70
$\bar{g}$ (mS/cm^2)	73	25	20	0.4

Note: g_{Na} and g_{HTK} values for soma are changed according to (Lorteije et al., 2009). They are set to 7.3 and 2, respectively (mS/cm2).

Table S3: Values of the subunit kinetics of MNTB principal cell

	m	h	l	r	n	p
k_α (ms^{-1})	76.4	5.33 x 10^{-4}	1.2	0.0438	0.2719	7.13 x 10^{-3}

η_α (mV^{-1})	0.037	-0.0909	0.0351	-0.0053	0.04	-0.1942
k_β (ms^{-1})	6.93	0.787	0.2248	0.0562	0.1974	0.0935
η_β (mV^{-1})	-0.043	0.0691	-0.0319	-0.0047	0	0.0058
Q_{10}	3	2.36	3	-	5	-

S.4 Kinetic Model of Synaptic Connection

Synaptic connections were modelled according to the equations and parameters described in (Graham et al., 2001). At a synapse, the arrival of an action potential (AP) initiates two different calcium transients, namely fast (C_{af} = 0.1mM for 1 ms) and slow (C_{as} = 0.01 mM for 2 ms) transients. The fast calcium transient initiates the opening of fast and slow gates. The fast gates go to open state quickly upon arrival of APs and close rapidly. On the other hand, slow gates open and close slowly so that a potentiation can be developed by the following AP. The probability of vesicle release (R) is defined by the product of the open states of slow and fast gates. During an AP, multiple active vesicles may be released independently. Once a vesicle is released, a 1 ms square pulse of neurotransmitter T is produced that drives an AMPA receptor-mediated EPSC at the soma (partially at the axon initial segment) of the principal cell. A 6-state gating model is used to simulate the AMPA receptor kinetics, composed of three closed states (C, C_1, C_2), fast and slow open states, and one desensitized state (Raman and Trussell, 1992). The EPSC at the receptor side is defined by

$$\mathrm{I} = \mathrm{g}(\mathrm{O}_1 + \mathrm{O}_2)(\mathrm{V} - \mathrm{E}_\mathrm{r}) \quad (36)$$

The replenishment of vesicles is mediated by a constant background replenishment from a large reserve pool, S, and the frequency-dependent replenishment is driven by the slow calcium transient. The replenishment rates are slow so that even sub-Hz stimulation can cause the depletion of the readily releasable vesicles.

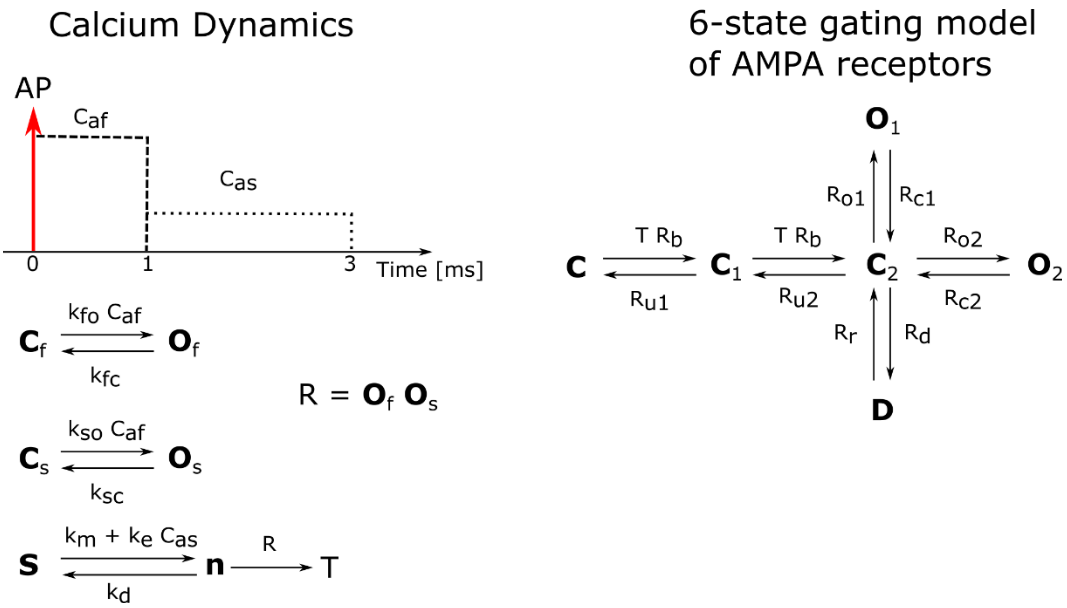

Figure S1: Kinetic models of synaptic transmission. A 6-state gating model is used to simulate the AMPA receptor kinetics, composed of three closed states (C, C_1, C_2), fast and slow open states, and one desensitized state. Adapted from (Graham et al., 2001).

**Table S4: Standard Kinetic Model parameters for Synaptic Connection (Adapted from** (Graham et al., 2001)**)**		
	Description	Value
Calcium Dynamics		
C_{af} (mM)	Fast calcium transient	0.1
C_{as} (mM)	Slow (residual) calcium transient	0.01
k_{fo} (1/ms-mM)	Fast gate opening	150
K_{fc} (1/ms)	Fast gate closing	30
k_{so} (1/ms-mM)	Slow gate opening	1
K_{sc} (1/ms)	Slow gate closing	0.1
k_m (1/s)	background replenishment rate	0.2
k_d (1/s)	background depletion of reserve pool	0.2
k_e (1/ms-mM)	rate of enhanced replenishment	8
6-state gating model of AMPA receptors		
R_b (1/ms-mM)	binding	13
R_{u1} (1/ms)	Unbinding of 1st site	0.3
R_{u2} (1/ms)	Unbinding of 2nd site	200
R_r(1/ms)	resensitization	0.02
R_d(1/ms)	desensitization	30
R_{o1} (1/ms)	Fast opening	100
R_{o2}(1/ms)	Slow opening	2
R_{c1} (1/ms)	Fast closing	2
R_{c2} (1/ms)	Slow closing	0.25
g (pS)	Maximal conductance of each receptor side	500
Er (mV)	Reversal potential	7

S.5 CHR2 Dynamics

The instantaneous rate of change of these states was defined by a set of rate equations, as given in (Grossman et al., 2011):

$$\frac{dO_1}{dt} = K_{a1}C_1 - (K_{d1} + e_{12})O_1 + e_{21}O_2 \tag{37}$$

$$\frac{dO_2}{dt} = K_{a2}C_2 + e_{12}O_1 - (K_{d2} + e_{21})O_2 \tag{38}$$

$$\frac{dC_2}{dt} = K_{d2}O_2 - (K_{a2} + K_r)C_2 \tag{39}$$

$$C_1 + C_2 + O_1 + O_2 = 1 \tag{40}$$

In this state model, only K_{a1} and K_{a2} rate constant dynamically change by the light illumination

$$K_{ai} = \begin{cases} \varepsilon_i\phi\left(1 - e^{-(t-t_{ON})/\tau}\right), & \text{Light ON} \\ \varepsilon_i\phi_0\left(e^{-(t-t_{OFF})/\tau} - e^{-(t-t_{ON})/\tau}\right), & \text{Light OFF} \end{cases} \tag{41}$$

where ε is the quantum efficiency of channelrhodopsin, ϕ is the photon flux per area, τ is the time constant of channel. Once O_1 and O_2 are determined, the photocurrent for illumination ON phase is calculated as:

$$i_{max} = (V - E_{ChR2})g_{ChR2} \tag{42}$$

where E_{ChR2} is the reversal potential (set to 0 mV) and g_{ChR2} is the channel conductance per area ($g_{ChR2} = g_1\rho_{ChR2}$).

After the illumination goes OFF, the ChR2 current shows an exponential decay which explained by a fast (i_{fast}) and slow (i_{slow}) component, given as

$$i = i_{slow}\, e^{-\Lambda_1(t-t_{OFF})} + i_{fast}\, e^{-\Lambda_2(t-t_{OOF})} \tag{43}$$

where Λ_1 and Λ_2 are decay factors (Table S5). The fast and slow components are defined by:

$$i_{fast} = i_{max}\frac{O_{10}\left[K_{d1} + (1-\gamma)e_{12,dark} - \Lambda_1\right] + O_{20}\left[\gamma(K_{d2} - \Lambda_1) - (1-\gamma)e_{21,dark}\right]}{\Lambda_2 - \Lambda_1} \tag{44}$$

$$i_{slow} = i_{max}\frac{O_{10}\left[\Lambda_2 - K_{d1} - (1-\gamma)e_{12,dark}\right] + O_{20}\left[(1-\gamma)e_{12,dark} + \gamma(\Lambda_2 - K_{d2})\right]}{\Lambda_2 - \Lambda_1} \tag{45}$$

where O_{10} and O_{20} are the fraction of open channels at the end of illumination ON phase, and γ is the ratio of the conductance of the two states O_2 and ($\gamma = g_2/g_1$)

**Table S5: Standard model parameters for Optical stimulation (Adapted from** (Foutz et al., 2012)**)**		
	Description	Value
Channelrhodopsin-2 properties		
K_{d1} (s^{-1})	Decay Rate	130
K_{d2} (s^{-1})	Decay Rate	25
Λ_1 (μs)	Decay factor	30
Λ_2 (μs)	Decay factor	150
e_{12} (s^{-1})	Transition rate: light	53
	Transition rate: dark	22
e_{21} (s^{-1})	Transition rate: light	23
	Transition rate: dark	11
K_r (s^{-1})	Recovery rate	0.4
g_1 (fS)	O1 state conductivity	50
g_2 (fS)	O2 state conductivity	2.5
σ_{ret} (μm^{-2})	Retinal cross section	1.2×10^{-8}
ε_1	Quantum efficiency	0.5
ε_2	Quantum efficiency	0.1
τ (ms)	ChR2 time constant	1.3
ρ_{ChR2}* (μm^{-2})	ChR2 density	130
Fiber optic-Tissue Properties		
NA_{fiber}	Optical fiber numerical aperture	0.37
μ_a (mm^{-1})	Absorbance coefficient, gray matter (Aravanis et al., 2007a)	0.125
	pedunculopontine nucleus (Yona et al., 2016)	0.06
μ_s (mm^{-1})	Scattering coefficient, gray matter (Aravanis et al., 2007a)	7.37
	pedunculopontine nucleus (Yona et al., 2016)	16.86
η_{tissue} (mm^{-1})	Tissue index of refraction	1.36

S.6 Extracellular Stimulation

The extracellular electric potential at $\mathbf{r} = (x, y, z)$ created by a point-like electrode with an excitation current of $\mathbf{I_e}$ and located at $\mathbf{r}_e = (x_e, y_e, z_e)$ in an infinite volume conductor with isotropic and homogeneous extracellular electrical conductivity σ_e is given as (Nunez and Srinivasan, 2006):

$$\phi_h(x, y, z, x_e, y_e, z_e) = \frac{I_e}{4\pi\sigma_e} \frac{1}{\sqrt{(x - x_e)^2 + (y - y_e)^2 + (z - z_e)^2}} \quad (46)$$

In *in vitro* brain slice experiments, the stimulation electrode is placed in between two planar interfaces at which the conductivity changes, i.e. the tissue-saline and tissue-diamond interfaces as shown in Fig. S2A. For this configuration, Eq. 46 has to be adapted to take into account the contribution of the conductivity changes. For that, we use the "method of image" in which the effect of a planar discontinuity in the conductivity is handled by placing virtual sources at the same distance on the opposite side of the boundaries (Gold et al., 2006; Ness et al., 2015). Considering a stimulation point electrode positioned at (x_e, y_e, z_e) inside a slice extending from $z = 0$ to $z = h$, the extracellular potential is given by (Ness et al., 2015)

$$\begin{aligned} \phi_e(x, y, z, x_e, y_e, z_e) &= \phi_h(x, y, z, x_e, y_e, z_e) \\ &+ \sum_{n=0}^{\infty} W_{TB}^n W_{TS}^n \big(W_{TS}\phi_h(x, y, z, x_e, y_e, -z_e + 2(n+1)h) + W_{TB}\phi_h(x, y, z, x_e, y_e, -z_e - 2nh)\big) \\ &+ \sum_{n=1}^{\infty} W_{TB}^n W_{TS}^n \big(\phi_h(x, y, z, x_e, y_e, z_e - 2nh) + \phi_h(x, y, z, x_e, y_e, z_e + 2nh)\big) \end{aligned} \quad (47)$$

where $W_{TB} = (\sigma_T - \sigma_B)/(\sigma_T + \sigma_B)$ and $W_{TS} = (\sigma_T - \sigma_S)/(\sigma_T + \sigma_S)$ are scaling factors depending on the conductivity of the tissue σ_T, the conductivity of saline σ_S, and the conductivity of the bottom layer σ_B. In our calculation, we used $\sigma_T = 0.3$ S/m (Hämäläinen et al., 1993) and $\sigma_S = 1.5$ S/m (Nunez and Srinivasan, 2006). The infinite series in Eq. 47 converges fast so that we approximated it by the first 10 terms (Gold et al., 2006).

The extracellular stimulation is implemented using the extracellular mechanism of NEURON (Carnevale and Hines, 2006). It allows changing the extracellular membrane voltage from ground to the level determined by Eq. 47. A simplified drawing is shown in Fig. S2B. In this case, the cable equation to compute the time courses of the transmembrane voltage $V_n = V_{i,n} - V_{e,n}$ will then be as follow

$$C_n \frac{dV_n}{dt} = \left[-I_{ion,n} + \frac{V_{n-1} - V_n}{R_{a,n-1/2}} + \frac{V_{n+1} - V_n}{R_{a,n+1/2}} - \frac{V_{e,n-1} - V_{e,n}}{R_{a,n-1/2}} + \frac{V_{e,n+1} - V_{e,n}}{R_{a,n+1/2}}\right] \tag{48}$$

where $V_{e,n}$ is the extracellular voltage of the compartment n and it is determined by Eq. 47, $I_{ion,n}$ is the ion channel current, C_n is the membrane capacitance and R_a is the axial resistance. Please see Fig. S12 for the simulation results for the extracellular electrical stimulation.

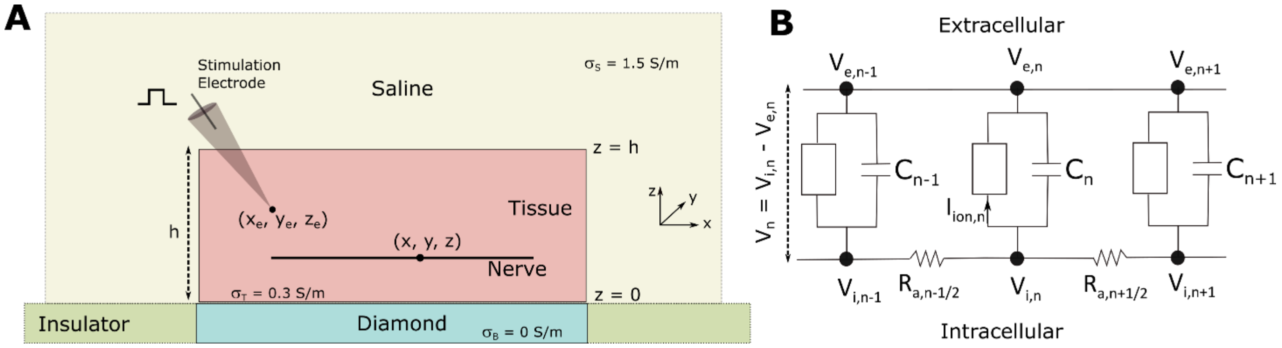

Figure S2: (A) Schematic image of the extracellular stimulation electrode embedded in brain tissue and surrounded by saline and an insulating bottom layer. **(B)** The compartment model to determine the transmembrane voltage $V_n = V_{i,n} - V_{e,n}$.

Supplementary Figures

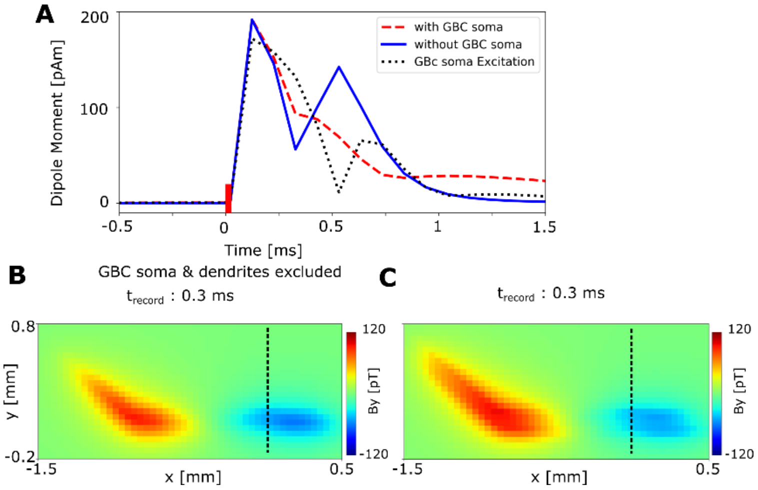

Figure S3: Contribution of the GBC soma and dendrites to the magnetic fields. **(A)** Dipole moment for three different scenarios: (1) Soma and dendrites of GB cells included in the simulations. (2) Without GBC soma and dendrites. In both cases, the stimulation is applied at the calyces of Held (as in Figure 6 in main text). (3) Last, the current stimulation is applied to the soma of GBC. **(B-C)** Up to a time point of t = 0.3 ms, the By-component (the strongest component) of the magnetic field is dominantly determined by APs traveling along the myelinated axonal pathway.

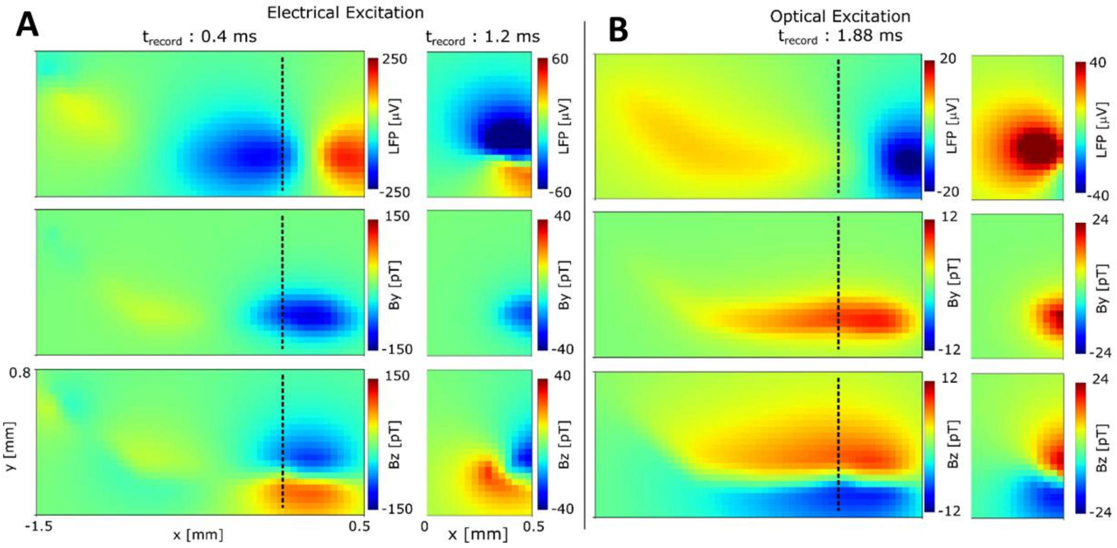

Figure S4: (A) Comparison of the contributions of the GB cells and the MNTB principal cells to the recorded fields, in response to an electrical stimulation of the GBC soma region. The simulated pathway consists of 300 GB cells distributed in a 300 µm thick slice, and 300 MNTB principal cells. Left column: LFP and magnetic fields at t = 0.4 ms after the start of the electrical pulse. Right column: The field distributions at t = 1.2 ms are shown for a field of view focused on MNTB region on the right side of the brain midline. The Bx-component of the magnetic field is not shown here since it is much weaker. **(B)** Magnetic fields contribution of 300 GB cells and MNTB principal cells at t = 1.88 ms after start of the light pulse (~1 W/mm^2 source irradiance). The spatial distributions are similar to the case of electrical stimulation. In contrast to electrical stimulation, however, the contribution of MNTB cells is more prominent.

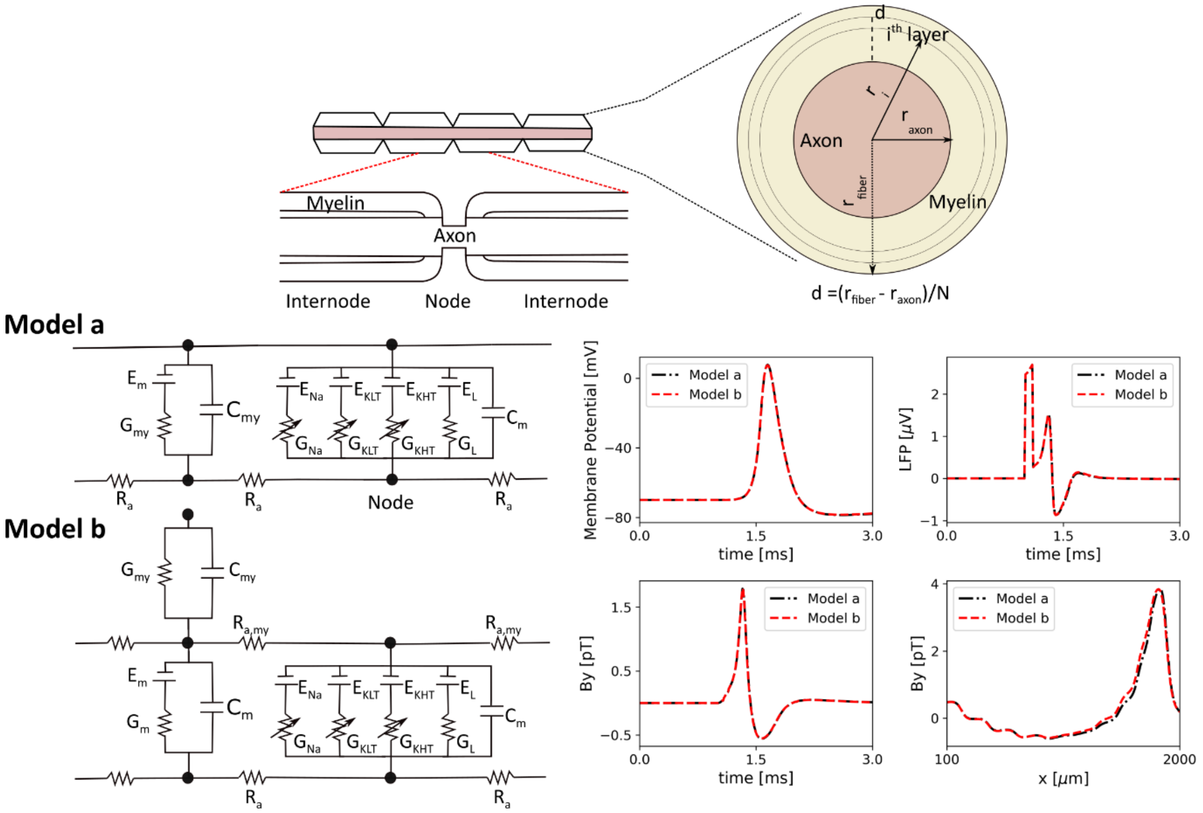

Figure S5: Multi-compartment diagram of a myelinated axon. Two different electrical models (Model B and C of (Richardson et al., 2000)) were simulated. The nodal membrane dynamics included high threshold (G_{KHT}) and low threshold (G_{KLT}) potassium, sodium (G_{Na}), and leakage (G_L) conductances in parallel with the nodal capacitance (C_m). The internodal segments were represented by a double cable structure of linear conductances with an explicit representation of the myelin sheath (G_{my} in parallel with C_{my}) and the internodal axolemma (G_m in parallel with C_m). E_m denotes the reversal potential of the membrane. The transmembrane potential and extracellular fields along 2 mm long myelinated axon are determined and given.

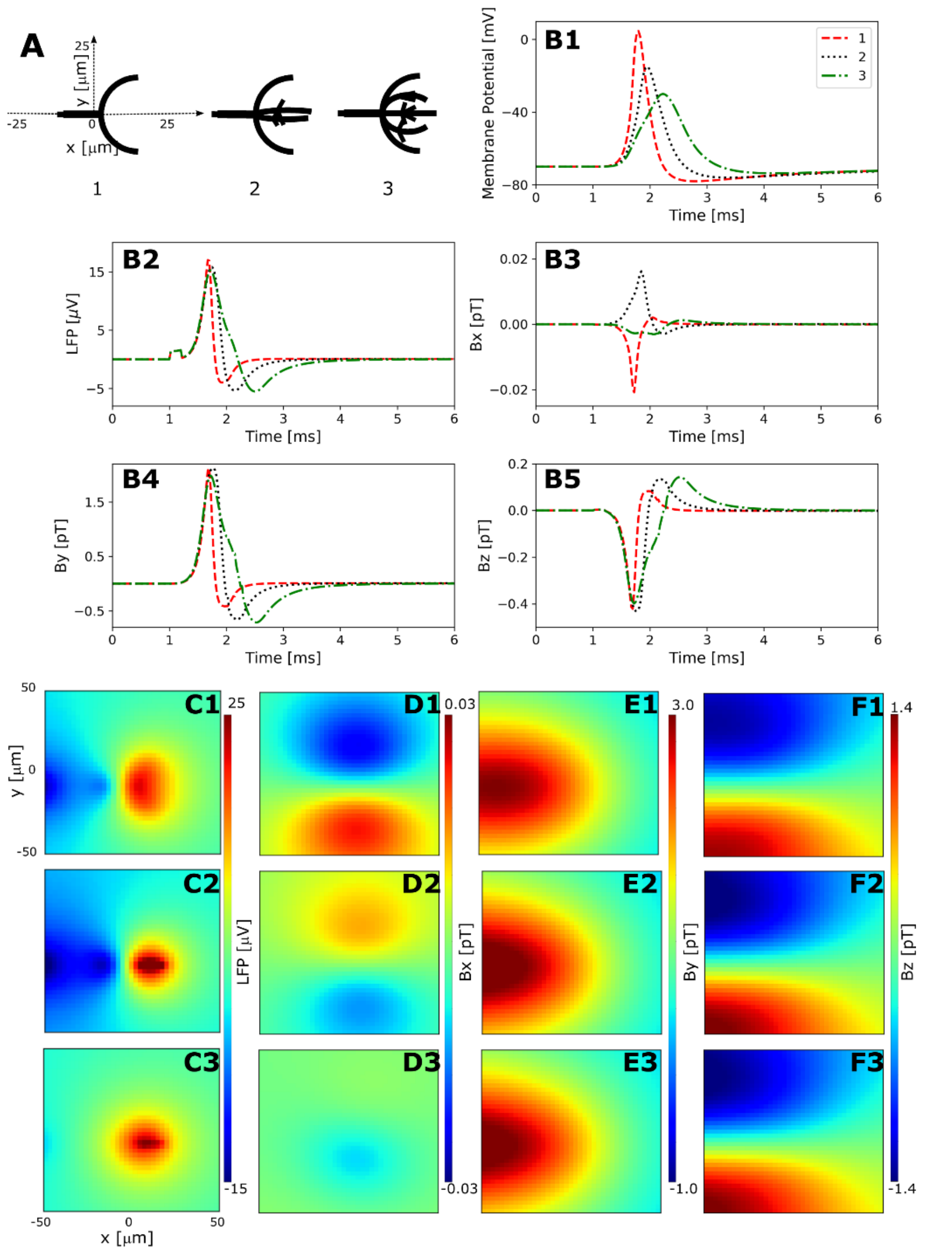

Figure S6: Influence of the calyx morphological structure on the extracellular fields. **(A)** In the first model, the calyx is approximated as a two quarter circles. In the second model, two quarters are added perpendicular to the initial calyx. At the center of each quarter circle, two stalks by length of 6 µm are placed at random orientations. In the third model, additional smaller two quarters oriented 45 degree from the slice plane are added to the structures of the second model. **(B1-B5)** The transmembrane potential at the calyx of Held and extracellular fields are given. Increasing the number of stalks and circles decreased the level of depolarization, and the APs became temporarily broadened. However, the extracellular field amplitudes did not change substantially. **(E-F)** The spatial distribution of the LFP and the magnetic field components at a surface with an area of 0.1 x 0.1 mm^2 at a distance of 50 µm. The surface was divided in a 40 x 40 grid and the fields are

visualized at the time points when they peak. The dominant magnetic field components, By and Bz, remain in the same range. Since the GBC axon was aligned along the y direction, the Bx component is negligibly weak for all cases.

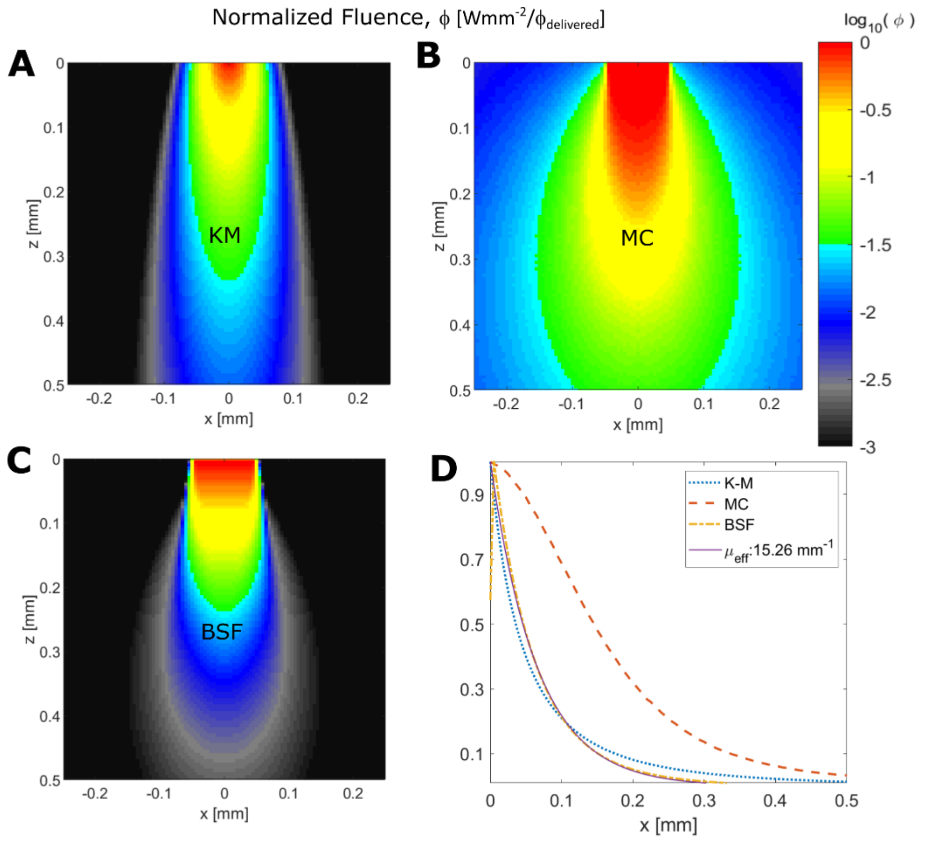

Figure S7: Comparison of different light simulation methods for stimulation of the pedunculopontine tegmental nucleus with experimental data. Tissue-scattering/absorption parameters: μ_s = 16.86 mm^{-1}, μ_a = 0.06 mm^{-1}, g = 0.88; Optical fiber parameters: NA = 0.22, diameter = 100 µm. **(A)** Kubelka-Monk (K-M) simulation (Mobley et al., 2014). **(B)** Monte-Carlo (MC) light simulation (Wang et al., 1995). **(C)** Beam spread function (BSF) method (Yona et al., 2016). **(D)** Transmission of light along the z-axis, comparing published experimental results (solid) given in (Al-Juboori et al., 2013) with the simulation results. In (Al-Juboori et al., 2013), the effective attenuation constant is determined as μ_{eff} = 15.26 mm^{-1}.

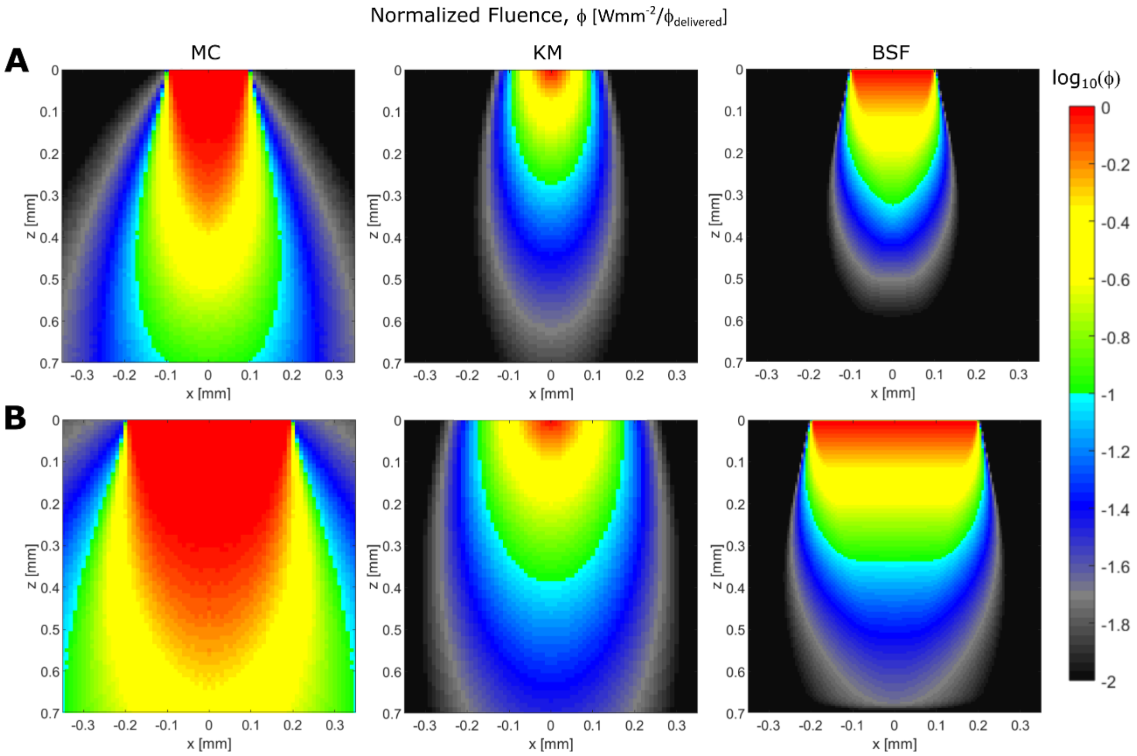

Figure S8: Comparison of different light simulation methods for gray matter. Tissue-scattering/absorption parameters: μ_s = 7.37 mm^{-1}, μ_a = 0.125 mm^{-1}, g = 0.91; Optical fiber parameters: NA = 0.37. **(A)** Fiber diameter is set to 200 µm for comparison with the measurement results given in (Aravanis et al., 2007b; Yizhar et al., 2011). In (Yizhar et al., 2011), the light intensity decreases to ~2% of its maximum at a distance of 0.5 mm from the fiber. This decrease is clearly underestimated in the MC simulations. **(B)** Optical diameter is set to 400 µm for comparison with the measurements given in (Gradinaru et al., 2009). The measured light density decreased to ~10% of its peak intensity at a distance of 0.4 mm from the fiber tip (Gradinaru et al., 2009). Again, this decrease is strongly underestimated in the MC simulations.

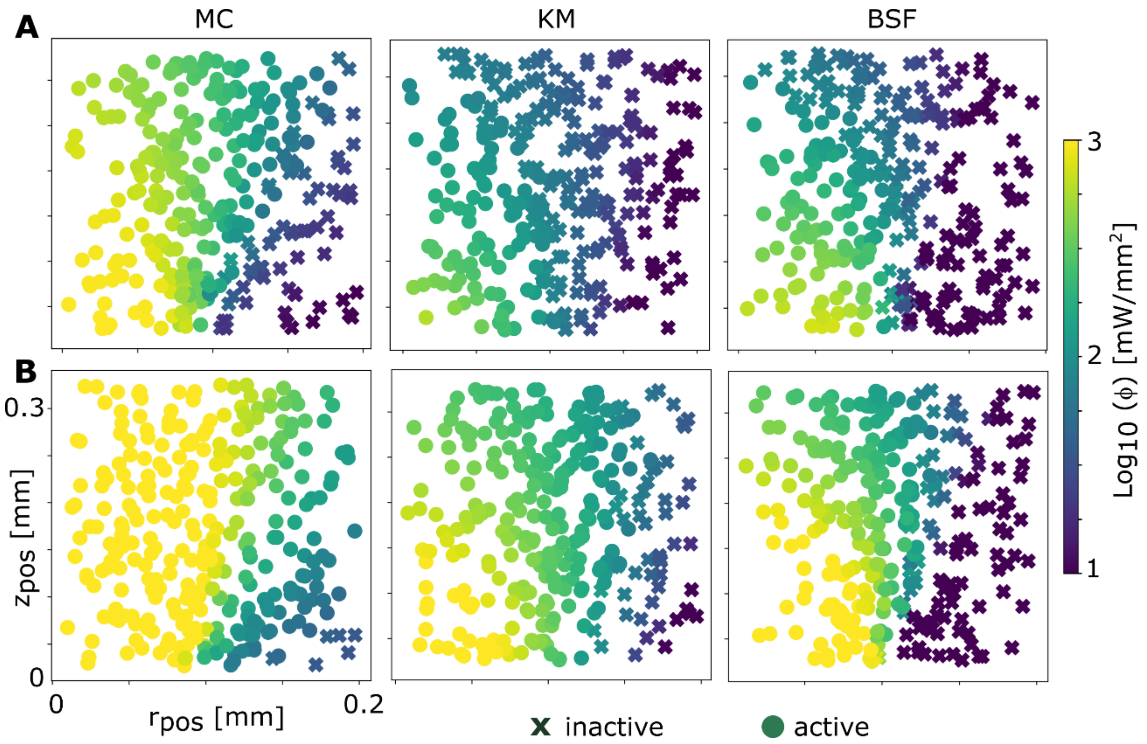

Figure S9: Comparison of different light simulation methods for gray matter. Tissue-scattering/absorption parameters: μ_s = 7.37 mm^{-1}, μ_a = 0.125 mm^{-1}, g = 0.91; Optical fiber parameters: NA = 0.37, diameter = 200 µm. The light intensities at the calyces with respect to their axial and radial distance from the center of light probe are plotted. 300 cells are uniformly distributed over the MNTB region in a 300 µm thick slice. The disk and square denote the spiking (active) and non-spiking (inactive) cells, respectively. **(A)** The source power at the fiber tip is set to 30 mW. **(B)** The source power at the fiber tip is set to 120 mW.

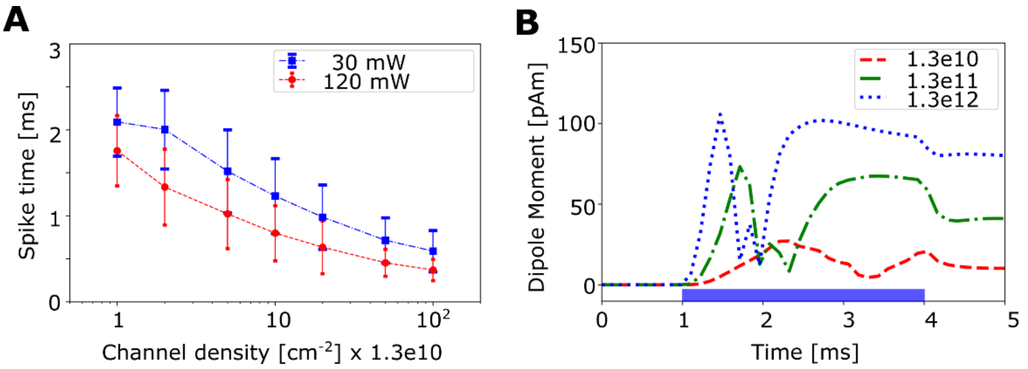

Figure S10: Effect of the ChR2 channel density on spike timing and the equivalent current dipole moment. Tissue-scattering/absorption parameters: μ_s = 16.86 mm^{-1}, μ_a = 0.06 mm^{-1}, g = 0.88. Optical fiber parameters: NA = 0.37, diameter = 200 µm. **(A)** The mean and standard deviation of the spiking time in calyces for 300 cells in the brainstem slice are shown for two different light levels. The channel density is varied between from 1.3e10 to 1.3e12 μm^{-2}, according to the two estimates reported in (Arlow et al., 2013). Increasing the channel density results in more synchronous and faster events. **(B)** Equivalent current dipole moments for three different channel densities under 120 mW light power at the probe tip. Since higher channel densities result in more synchronous spikes, the ECD increases.

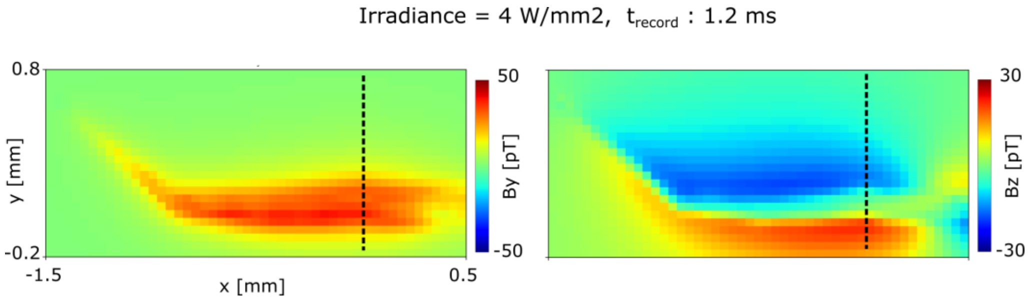

Figure S11: Simulation results showing the spatial distributions of the extracellular electric potential and neural magnetic fields of the auditory GBC-MNTB pathway in response to an optical stimulation (4 W/mm^2 irradiance at the source) of the MNTB region. LFP and magnetic fields at t = 1.2 ms after the start of the electrical pulse. The field distributions are in accordance with an axial current flow from the last quarter of the axonal pathway to the MNTB region, caused by action potentials traveling antidromically along the pathway.

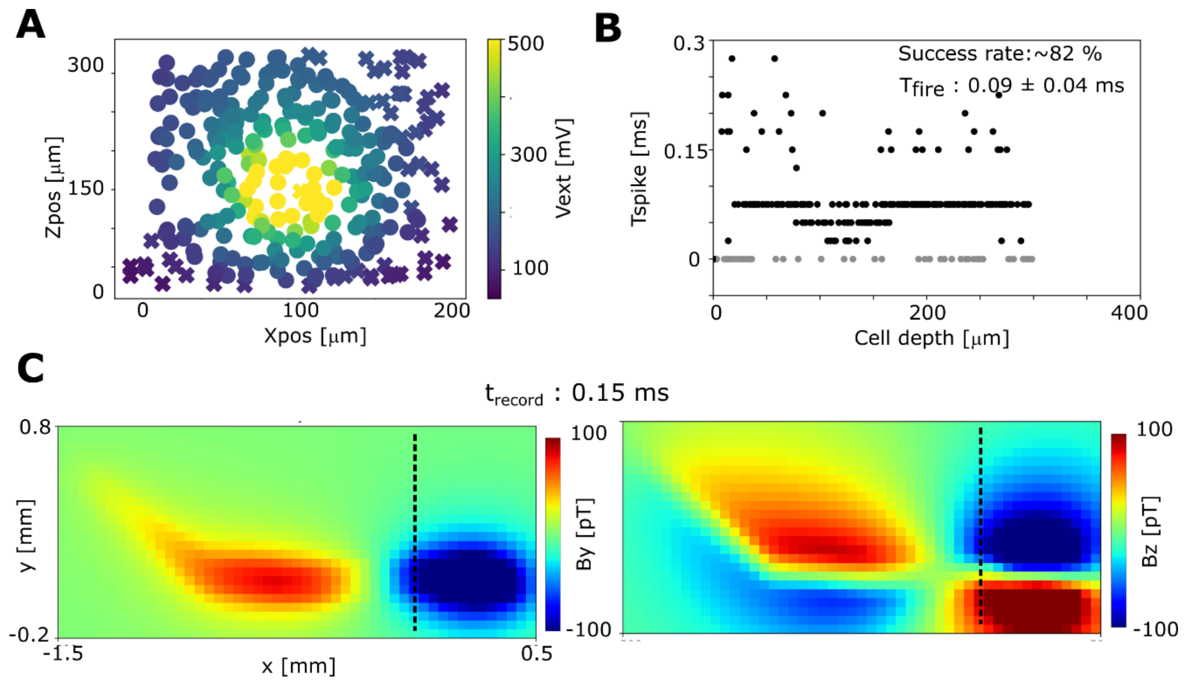

Figure S12: Extracellular electrical stimulation with a monopolar electrode placed in the center of the MNTB region. 300 cells are uniformly distributed over the MNTB region in a 300 µm thick slice, and the electrode tip was placed at a depth of $z_e = 150$ µm. Stimulation was performed with a current amplitude of 100 µA and a pulse duration of 100 µs. **(A)** The extracellular voltage at the calyces of Held with respect to their z and x position from the center of MNTB region. The disk and square denote the spiking (active) and non-spiking (inactive) cells, respectively. Around 82% of the axons of the GB cells are excited by the extracellular current pulse. **(B)** Spike times of the 300 cells in the brainstem slice stimulated. Inactive cells are plotted as grey dots, activated as black dots. Most cells were excited at 1st or 2nd internode counted from the calyx. The mean firing times at the (arbitrarily selected) 6th node were 0.09 ms, with a low standard deviation of ± 0.04 ms (SD), demonstrating the good synchrony of the APs. **(C)** Magnetic fields at t = 0.15 ms after the start of the stimulation. During stimulation, the anode electrode hyperpolarized the MNTB region. As a result, the cells are recovering from that hyperpolarization after stimulation end which causes a large current flow. The neural magnetic field strength due to the APs traveling along the axon pathway reaches ~100 pT.

References

Al-Juboori, S. I., Dondzillo, A., Stubblefield, E. A., Felsen, G., Lei, T. C., and Klug, A. (2013). Light Scattering Properties Vary across Different Regions of the Adult Mouse Brain. *PLoS One* 8, e67626. doi:10.1371/journal.pone.0067626.

Aravanis, A. M., Wang, L.-P., Zhang, F., Meltzer, L. A., Mogri, M. Z., Schneider, M. B., et al. (2007a). An optical neural interface: in vivo control of rodent motor cortex with integrated fiberoptic and optogenetic technology. *J. Neural Eng.* 4, S143–S156. doi:10.1088/1741-2560/4/3/S02.

Aravanis, A. M., Wang, L., Zhang, F., Meltzer, L. A., Mogri, M. Z., Schneider, M. B., et al. (2007b). An optical neural interface: in vivo control of rodent motor cortex with integrated fiberoptic and optogenetic technology. *J. Neural Eng.* 4, S143–S156. doi:10.1088/1741-2560/4/3/S02.

Arlow, R. L., Foutz, T. J., and McIntyre, C. C. (2013). Theoretical principles underlying optical stimulation of myelinated axons expressing channelrhodopsin-2. *Neuroscience* 248, 541–551. doi:10.1016/j.neuroscience.2013.06.031.

Carnevale, N. T., and Hines, M. L. (2006). *The NEURON Book*. Cambridge: Cambridge University Press doi:10.1017/CBO9780511541612.

Ford, M. C., Alexandrova, O., Cossell, L., Stange-Marten, A., Sinclair, J., Kopp-Scheinpflug, C., et al. (2015). Tuning of Ranvier node and internode properties in myelinated axons to adjust action potential timing. *Nat. Commun.* 6, 8073. doi:10.1038/ncomms9073.

Foutz, T. J., Arlow, R. L., and McIntyre, C. C. (2012). Theoretical principles underlying optical stimulation of a channelrhodopsin-2 positive pyramidal neuron. *J. Neurophysiol.* 107, 3235–3245. doi:10.1152/jn.00501.2011.

Gold, C., Henze, D. A., Koch, C., and Buzsáki, G. (2006). On the Origin of the Extracellular Action Potential Waveform: A Modeling Study. *J. Neurophysiol.* 95, 3113–3128. doi:10.1152/jn.00979.2005.

Gradinaru, V., Mogri, M., Thompson, K. R., Henderson, J. M., and Deisseroth, K. (2009). Optical Deconstruction of Parkinsonian Neural Circuitry. *Science (80-.).* 324, 354–359. doi:10.1126/science.1167093.

Graham, B. P., Wong, A. Y. C., and Forsythe, I. D. (2001). A computational model of synaptic

transmission at the calyx of Held. *Neurocomputing* 38–40, 37–42. doi:10.1016/S0925-2312(01)00476-3.

Grossman, N., Nikolic, K., Toumazou, C., and Degenaar, P. (2011). Modeling study of the light stimulation of a neuron cell with channelrhodopsin-2 mutants. *IEEE Trans. Biomed. Eng.* 58, 1742–1751. doi:10.1109/TBME.2011.2114883.

Hämäläinen, M., Hari, R., Ilmoniemi, R. J., Knuutila, J., and Lounasmaa, O. V. (1993). Magnetoencephalography—theory, instrumentation, and applications to noninvasive studies of the working human brain. *Rev. Mod. Phys.* 65, 413–497. doi:10.1103/RevModPhys.65.413.

Lorteije, J. A. M., Rusu, S. I., Kushmerick, C., and Borst, J. G. G. (2009). Reliability and Precision of the Mouse Calyx of Held Synapse. *J. Neurosci.* 29, 13770–13784. doi:10.1523/JNEUROSCI.3285-09.2009.

Mobley, J., Vo-Dinh, T., and Tuchin, V. V. (2014). “Optical properties of tissue,” in *Biomedical Photonics Handbook, 3 Volume Set,* ed. T. Vo-Dinh (CRC Press). doi:10.1201/b17290.

Ness, T. V, Chintaluri, C., Potworowski, J., Łęski, S., Głąbska, H., Wójcik, D. K., et al. (2015). Modelling and Analysis of Electrical Potentials Recorded in Microelectrode Arrays (MEAs). *Neuroinformatics* 13, 403–426. doi:10.1007/s12021-015-9265-6.

Nunez, P. L., and Srinivasan, R. (2006). *Electric Fields of the Brain*. Oxford University Press doi:10.1093/acprof:oso/9780195050387.001.0001.

Raman, I. M., and Trussell, L. O. (1992). The kinetics of the response to glutamate and kainate in neurons of the avian cochlear nucleus. *Neuron* 9, 173–186. doi:10.1016/0896-6273(92)90232-3.

Richardson, A. G., McIntyre, C. C., and Grill, W. M. (2000). Modelling the effects of electric fields on nerve fibres: Influence of the myelin sheath. *Med. Biol. Eng. Comput.* 38, 438–446. doi:10.1007/BF02345014.

Rothman, J. S., and Manis, P. B. (2003). Kinetic Analyses of Three Distinct Potassium Conductances in Ventral Cochlear Nucleus Neurons. *J. Neurophysiol.* 89, 3083–3096. doi:10.1152/jn.00126.2002.

Wang, L.-Y., and Kaczmarek, L. K. (1998). High-frequency firing helps replenish the readily releasable pool of synaptic vesicles. *Nature* 394, 384–388. doi:10.1038/28645.

Wang, L., Jacques, S. L., and Zheng, L. (1995). MCML-Monte Carlo modeling of light transport in multi-layered tissues. *Comput. Methods Programs Biomed.* doi:10.1016/0169-2607(95)01640-F.

Yizhar, O., Fenno, L. E., Davidson, T. J., Mogri, M., and Deisseroth, K. (2011). Optogenetics in Neural Systems. *Neuron* 71, 9–34. doi:10.1016/j.neuron.2011.06.004.

Yona, G., Meitav, N., Kahn, I., and Shoham, S. (2016). Realistic Numerical and Analytical Modeling of Light Scattering in Brain Tissue for Optogenetic Applications. *eNeuro* 3, 1–9. doi:10.1523/ENEURO.0059-15.2015.
